# Solvatochromic Polarity, Physicochemical Properties, and Spectral Analysis of New Triple NADES-Based on Urea–Glycerol

**DOI:** 10.3390/molecules31020233

**Published:** 2026-01-09

**Authors:** Sezan Ahmed, Dimitar Bojilov, Ginka Exner, Soleya Dagnon, Stanimir Manolov, Iliyan Ivanov

**Affiliations:** 1 Department of Organic Chemistry, Faculty of Chemistry, University of Plovdiv, 24 Tsar Assen Str., 4000 Plovdiv, Bulgaria; nsahmed998@gmail.com (S.A.); solbono@abv.bg (S.D.); manolov@uni-plovdiv.bg (S.M.); iiiliyan@abv.bg (I.I.); 2 Department of Physics, Faculty of Physics and Technology, Plovdiv University Paisii Hilendarski, 4000 Plovdiv, Bulgaria; exner@uni-plovdiv.bg

**Keywords:** natural deep eutectic solvent (NADES), polarity, solvatochromism, Kamlet–Taft, contact angle, surface tension, density, refractive index, green solvents

## Abstract

In the present study, ten type-V natural deep eutectic solvents (NADESs) were synthesized and comprehensively characterized, based on urea as a hydrogen-bond acceptor and three different groups of donors—glycerol, organic carboxylic acids, and carbohydrates. Their physicochemical parameters, spectral characteristics (FTIR), surface tension, and solvatochromic properties were determined using Nile Red, betaine 30, and Kamlet–Taft parameters. The densities of the systems (1.243–1.361 g/cm^3^) and the high values of molar refraction and polarizability indicate the formation of highly organized hydrogen-bonded networks, with the incorporated carboxyl and hydroxyl groups enhancing the structural compactness of the NADES. Surface tension varied significantly (46.9–80.3 mN/m), defining systems with low, medium, and high polarity. Solvatochromic analysis revealed high E_NR_, E_T_(30), and *E_T_^N^* values, positioning all NADES as highly polar media, comparable or close to water, but with distinguishable H-bond donating/accepting ability depending on the third component. The normalized Kamlet–Taft parameters show that the NADES cover a broad solvent spectrum—from highly H-bond accepting to strongly H-bond donating or dipolar systems—highlighting the potential for fine-tuning the solvent according to target applications. The obtained results highlight the applicability of these NADESs as green, tunable media for the extraction and solvation of bioactive compounds.

## 1. Introduction

The development of green chemistry as a concept is focused on the creation of safe and environmentally sustainable chemical processes involving the use of non-toxic reagents, optimized conditions, and renewable energy. The primary objective is to minimize the ecological impact of the chemical industry [1,2,3]. One of the twelve principles of green chemistry underscores the utilization of environmentally compatible solvents, a factor that has precipitated an increased interest in sustainable alternatives [1]. Among the most significant innovations in this field are deep eutectic solvents (DESs)—mixtures of two or more components that form stable hydrogen bonds and form a eutectic system at a certain molar ratio [4]. The term “eutectic” is derived from the Greek εὔτηκτος, signifying “easily melted,” with the eutectic point denoting the composition and temperature at which the mixture melts at the lowest possible temperature. The definition of DES remains a subject of debate, as its definition is often similar to that of ionic liquids [5,6].

Initially, DESs were considered a subclass of ionic liquids (ILs) due to functional similarities. Today, DESs are fundamentally distinguished by their composition. While ILs consist of pure, synthetically prepared organic cations and anions that are liquid below 100 °C [7,8,9], DESs comprise hydrogen bond donors and acceptors (HBDs and HBAs). The interaction between HBDs and HBAs in DESs lowers the lattice energy and melting point, with the effect depending on the strength of the hydrogen bonds and the structure of the components used [8,10].

In 2003, Abbott introduced the term “deep eutectic solvent” for mixed systems formed by hydrogen bonds between polar components [11]. DES can be categorized into five primary classifications. Type I is defined as a quaternary ammonium salt in combination with a metal chloride (HBD). Type II is analogous to type I, but with the addition of hydrated metal halides. Type III comprises ammonium salt in combination with organic HBD, encompassing carboxylic acids, amides, and polyols. The fourth type is characterized by the presence of metal halides in conjunction with organic ligands. Type V is characterized by the presence of entirely nonionic components, which are linked by hydrogen bonds [4,8,11,12]. In addition to this classification, terms such as natural, synthetic, acidic, basic, ionic, nonionic, hydrophilic, hydrophobic, therapeutic, or polymeric DESs are also commonly used [12,13].

Natural deep eutectic solvents (NADESs) are characterized by high biocompatibility and low toxicity. They are composed of metabolic molecules, including organic acids, amino acids, polyols, sugars, and choline chloride derivatives [4,13]. The first description of these compounds was by Choi et al. in 2011, who suggested that NADESs could be the “missing link” in explaining cellular metabolism, allowing the high solubility of hydrophobic compounds in cells [7,14]. It is evident that the composition of a NADES is highly diverse, and consequently, they are classified into five distinct groups: ionic liquids; neutral; neutral with acids; neutral with bases; and containing amino acids [15,16].

Therapeutic deep eutectic solvents (THEDESs), which incorporate active pharmaceutical ingredients, significantly enhance drug bioavailability and transdermal delivery while reducing cytotoxicity [13,17,18,19,20,21,22,23,24,25]. In parallel, poly-quasi-eutectic solvents (PQESs) combine polymer chemistry with eutectic principles, serving as a transitional phase toward ionic liquids and expanding the possibilities for dissolving inorganic materials [6,26,27].

DESs surpass conventional solvents with their unique properties, necessitating detailed characterization using physicochemical methods. The distinctive physicochemical properties of DESs depend directly on the nature of the HBA/HBD components and their hydrogen bonding: density increases in dense networks and ionic structures but decreases with long alkyl chains [12,28,29,30,31,32,33]. Viscosity is determined by molecular complexity and is highly sensitive to temperature and humidity [11,34,35,36]. Polarity ranges between that of methanol and water and is governed by the structure of the HBD [28,37,38,39], while surface tension and refractive index reflect the structural organization and polarizability of the components, exhibiting characteristic temperature-dependent behavior [40,41,42,43,44,45,46,47,48].

The literature reports type III DESs, which, due to their unique nature, are widely applied for the extraction of bioactive compounds [36,39,49,50,51,52,53,54,55,56,57,58,59,60,61,62,63,64,65,66,67,68,69,70,71,72,73]. Studies on ternary DESs indicate that the inclusion of additional components can effectively reduce viscosity compared to binary DESs [74].

This comprehensive investigation was conducted on a series of novel triple urea-based natural deep eutectic solvents (NADESs), a class of solvents whose properties have not been previously documented. Importantly, because these systems consist of neutral molecules, they do not conform to the conventional definition of ionic liquids. The primary objective of this study was to elucidate their fundamental physicochemical and spectral characteristics. We systematically determined their empirical polarity using a suite of solvatochromic probes, including Betaine 30, Nile Red (NR), 4-nitroanisole (ANS), and 4-nitrophenol (NP). Furthermore, the interfacial properties were characterized by measuring the surface tension (γ), specifically resolving it into its dispersion ($\gamma^{d}$) and polar ($\gamma^{p}$) components of surface energy. Currently, quantitative data on these components are unavailable for triple urea-based NADESs, preventing a rigorous assessment of their intrinsic adhesive and cohesive properties. The findings from this research provide critical data for establishing the polarity of these NADESs relative to established reference solvents, thereby facilitating their informed selection for various chemical and separation applications.

## 2. Results and Discussion

Unlike the widely studied type-III DES based on choline chloride, this study focuses on a type-V DES. This choice was motivated by the need for a fully molecular environment to enhance the solubility/interaction with hydrocarbons, which differs significantly from the ionic nature of salt-based eutectic mixtures.

In the present study, 10 urea-based NADES—one double and nine triple NADES were obtained.

It has been found that the binary mixture of urea and glycerol reaches its eutectic point at a molar ratio of 1:2, which determines its liquid state at room temperature [49]. When a third component (di-tricarboxylic acids and sugars) is introduced, a new ratio of 1:6:1 was experimentally defined, corresponding to a new eutectic point specific to the ternary system (Table 1). This composition provides the maximum decrease in the melting temperature compared to the individual components, which guarantees the stability of the liquid phase under operating conditions. Thus, the fixed molar ratio optimizes the balance of molecular interactions and provides the necessary rheological and adhesive characteristics of the resulting type-V NADES.

### 2.1. Physico-Chemical Properties of NADESs

#### 2.1.1. Density

The density of the solvents studied varied within the range 1.243–1.361 g/cm^3^ (Table 2). The lowest density was observed in the base solvent urea–glycerol NADES 1 (1.243 g/cm^3^), while all the other solvents had a higher density than NADES 1, water, and 70% MeOH. The density of solvents containing carbohydrates remains within narrow limits, whereas the density of DESs containing acids increases with the number of carboxyl and hydroxyl groups in the donor. This clearly demonstrates that the presence of these groups leads to the formation of a tighter, more organized hydrogen network, enabling denser packing of molecules within a given volume.

#### 2.1.2. Refractive Index

As expected, there is a significant difference between NADESs and conventional solvents in terms of molar refraction (***R_m_***) and polarizability coefficient (***δ***) values. The ***R_m_*** of NADES is 3–6 times higher than that of water and 70% methanol (Table 2). This directly indicates the significantly higher total electronic polarizability of NADESs, which measures the ability of molecular electron clouds to deform under the influence of an electric field (including light). The polarizability (***δ***), calculated from ***R_m_***, follows the same trend. In the NADES series, the ***R_m_*** and ***δ*** values increase as the size and complexity of the second HBD increase. The lowest values are observed in NADESs without a second HBD (NADES 1) or dicarboxylic acids (NADESs 3 and 4), while the highest values are obtained in NADESs containing carbohydrates (NADES 10; ***R_m_*** = 22.49 cm^3^/mol and ***δ*** = 8.92 × 10^−24^ cm^3^/mol) (Table 2). This clearly demonstrates that the polarizability of NADESs can be “predicted” by selecting a suitable second HBD. Internal pressure (***P_int_***) reflects total cohesive energy in the liquid. The data show that ***P_int_*** is 2.5 to 3.5 times lower than that of water (395 MPa) and significantly lower than that of 70% methanol (240 MPa) (Table 2). All NADESs are within a narrow range of 117 to 138 MPa. Of particular significance is the strong correlation between ***R_m_*** and ***P_int_*** (r = −0.9423) and between ***δ*** and ***P_int_*** (r = −0.9423). As the molar refraction (and polarizability) of the NADES increases, their internal pressure decreases. This phenomenon can be attributed to the observation that second HBDs with high polarizability (such as sugars) have been shown to enhance dispersion forces (London forces) within the solvent. Despite the weak dispersion forces, their accumulation in large, polarizable molecules can stabilize the system, manifesting as a decrease in cohesive energy, as reflected in ***P_int_***. This makes the NADES less “structured” than water, with weaker hydrogen networks, which explains their good ability to dissolve both polar and nonpolar compounds. The free molar volume (***f*_m_**), which characterizes the “empty” space in the liquid, is significantly higher in the NADES compared to water and methanol, which further supports the claim of their lower cohesive density (Table 2).

### 2.2. IR Spectral Characteristic of NADES

The ten analyzed solvent systems (NADESs) were grouped into three categories for systematic comparison and evaluation of the influence of the third component on the structure and spectral profile. This included a two-component reference system: Urea:Glycerol (1:2), three-component systems with carbohydrates:Urea:Glycerol. The following substances were analyzed: glucose, xylose, ribose, fructose (1:6:1), and sucrose in a ratio of 1:6:0.5, as well as three-component systems with carboxylic acids, namely urea and glycerol, as well as citric, malonic, maleic, and tartaric acids (1:6:1). This division facilitates a comparative analysis between the fundamental binary system and the diverse three-component systems. For pure urea, the characteristic amide bands—amide I (1673 cm^−1^) and amide II (1623 cm^−1^) (Figure S1)—were observed to shift to lower values in all NADES. The amide I band was found to shift to 1660–1664 cm^−1^, and the amide II band to 1626–1632 cm^−1^ (Figures S1–S10). This shift signifies that the carbonyl group of urea functions as an effective acceptor, actively participating in the formation of hydrogen bonds. Evidence for the formation of NADESs is found in the region of O–H and N–H valence vibrations at 3600–3100 cm^−1^. In all studied systems, a broad, intense, and asymmetric absorption band is observed. This band is the result of the overlap of the valence vibrations of all O–H groups (from glycerol, carbohydrates, and acids) and N–H groups (from urea), which participate as donors and acceptors of hydrogen bonds (Figures S1–S10). The pronounced broadening of the spectrum is a key spectral marker for the complex, intermolecular hydrogen-bonded network. NADESs containing –COOH groups exhibit a distinct spectral fingerprint. A distinct absorption band is observed in the range 1709–1728 cm^−1^, corresponding to the valence vibration of the carboxyl group C=O (Figures S2–S5). The bands for the different acids are, respectively, for citric acid: 1719 cm^−1^ (Figure S2), malonic acid: 1715 cm^−1^ (Figure S3), maleic acid: 1709 cm^−1^ (Figure S4), tartaric acid: 1728 cm^−1^ (Figure S5). The presence of this carbonyl band, as well as a broad O–H/N–H band in the range from 3338 to 3348 cm^−1^, confirms the participation of carboxyl groups as both donors and acceptors in hydrogen bonds and indicates greater structural complexity.

The three-component carbohydrate systems exhibit a high degree of structural similarity, attributable to the presence of numerous –OH groups (Figures S6–S10). In addition to the broad O–H/N–H band at 3335–3440 cm^−1^, a characteristic shift is observed in the amide region at 1660–1630 cm^−1^, indicating that hydrogen bonds are formed between the C=O group of urea and the hydroxyl groups of carbohydrates and glycerol. A thorough analysis of the fingerprint zone (1662–1626 cm^−1^) was conducted, which revealed a general tendency for broadening and mutual overlap of the C–O and C–N valence vibrations. In the fingerprint region (1200–900 cm^−1^), a slight broadening and change in the shape of the C–O/C–O–C vibrations were observed, indicating the replacement of the internal H bonds of monosaccharides with intermolecular H bonds in the liquid NADES system (Figures S6–S10). The presence of repeatable signals at 1110, 1040, and 993 cm^−1^ in the majority of samples is attributed to overlapping C–O and N–C–N deformation vibrations. Consequently, it can be concluded that NADESs derived from urea, glycerol, carboxylic acids, and carbohydrates are obtained through the formation of intermolecular hydrogen bonds between the hydrogen bond donor and acceptor rather than as a consequence of a chemical reaction between the components.

### 2.3. Contact Angle and Surface Tension

#### 2.3.1. General Information

Investigations on surface tension of deep eutectic solvents (NADESs) play a crucial role in understanding their interfacial behavior, leading to potential applications as extract agents, catalysts, reaction media, etc. [75,76,77]. In this respect, one has to evaluate the surface tension (γ), together with its polar (γᵖ) and dispersive (γᵈ) parts, as well as polarity (P), defined as the ratio γᵖ/γᵈ. The dispersive part is related to Lifshitz–van der Waals type of interactions, whereas the polar part is discussed in terms of Lewis acid-based interaction [78]. The parameters γᵈ and γᵖ correspond to cohesion and adhesion of NADES as well, at different interfaces, and define NADES stability, wettability, and surface energy. When the cohesive forces dominate, the contact angle of NADES, ***θ***, is higher than 90°, indicating poor wettability. On the contrary, a lower contact angle corresponds to a good adhesion of NADES and therefore a good wettability.

#### 2.3.2. Theoretical Background

The contact angle, ***θ***, of a liquid onto a solid surface can be quantified at a three-phase contact point of a small sessile liquid drop in thermal equilibrium on a horizontal surface. The phases in contact are the test liquid, the gaseous phase (air and/or liquid vapor), and the solid. When the goal is to investigate the surface tension of a liquid, such as a DES, several methods are applicable [78]: the capillary-rise method; the maximum-bubble-pressure method; the pending-drop-detachment-from-a-tube method; ring- or plate-rise methods; and sessile, hanging-drop, or bubble methods.

The main advantage of the sessile drop method is that one can derive not only the surface tension but its polar and dispersive parts as well. This method is usually applied for solid-surface investigations. Based on *γ* measurements of at least two different test liquids, with known surface tensions, one can estimate the surface tension of the solid. Most commonly used liquids are distilled water (DW, a polar liquid, ${\gamma_{DW}}^{p}=51\;mN/m$ and ${\gamma_{DW}}^{d}=21.8\;mN/m$) and methylene iodine (MI, a non-polar liquid, ${\gamma_{MI}}^{p}=0\;mN/m$ and ${\gamma_{MI}}^{d}=50.8\;mN/m$), although glycerol, formamide, ethylene glycol, *n*-hexane, and other liquids are also good choices [79,80,81,82]. Considering the forces’ balance at the three-phase contact point, one gets the following (Figure S11):

(1) $$\gamma_{\mathrm{sl}}=\gamma_{\mathrm{sv}}+\gamma_{\mathrm{lv}}\cos\theta$$

Taking into account that

(2) $$\gamma =\gamma^{d}+\gamma^{p}$$

Equation (1), with the help of Equation (2), can be rewritten [83,84,85,86] as

(3) $$\frac{\left(1+cos\theta \right)}{2}\frac{\gamma_{l}}{\sqrt{\gamma_{l}^{d}}}=\sqrt{\gamma_{s}^{p}}\sqrt{\frac{\gamma_{l}^{p}}{\gamma_{l}^{d}}}+\sqrt{\gamma_{s}^{d}},\;\;\;\;$$

where γ_l_, γ_l_^d^, and γ_l_^p^ are the total, the dispersive, and the polar part of the surface tension of the test liquids, respectively; γ_s_, γ_s_^d^, and γ_s_^p^ are the total, the dispersive, and the polar part of the surface tension of the solid surfaces, respectively; ***θ*** is the contact angle at the solid–liquid interface.

Following the same approach, Equation (3) can be reformulated with respect to an unknown liquid surface tension, in our case, NADES, when the γ of a solid surface is known. The surface tension of NADES can be evaluated by measuring the contact angle and analyzing the following equation:

(4) $$\frac{\left(1+\cos\theta \right)}{2\sqrt{\gamma_{s}^{p}}}=\left(\sqrt{\frac{\gamma_{l}^{d}}{{\gamma_{l}}^{2}}}\right)\frac{\sqrt{\gamma_{s}^{d}}}{\sqrt{\gamma_{s}^{p}}}+\left(\sqrt{\frac{\gamma_{l}^{p}}{{\gamma_{l}}^{2}}}\right)$$

from a linear regression of this linear dependence, where $y=0.5\left(1+\cos\theta \right)\sqrt{\gamma_{s}^{p}}$ and $x=\sqrt{\gamma_{s}^{d}/\gamma_{s}^{p}}$, the slope $a=\sqrt{\gamma_{l}^{d}/{\gamma_{l}}^{2}}$ and the intersect $b=\sqrt{\gamma_{l}^{p}/{\gamma_{l}}^{2}}$ can be derived. Finally, both parts of the surface tension of DES can be obtained as

(5) $$\gamma_{l}^{p}=\frac{b^{2}}{{\left(a^{2}+b^{2}\right)}^{2}}$$

(6) $$\gamma_{l}^{d}=\frac{a^{2}}{{\left(a^{2}+b^{2}\right)}^{2}}$$

The results from the linear regression of Equation (4) and the calculations of the surface tensions of NADES, based on Equations (5) and (6), are given in Table 3. The square factor was excellent, at least R^2^ = 0.99977, showing the reliability of the obtained results.

#### 2.3.3. Surface Tension of NADESs

The surface tension of the investigated NADESs varies from 46.9 to 80.3 mN/m, spreading from low-surface organic liquids to highly polar water systems (Table 3). The lowest value (46.9 mN/m) was measured for NADES 4, where the polarity is the lowest as well, being 0.04, demonstrating the predominance of dispersion forces and weak cohesion. In contrast, the highest surface tension was observed for NADES 2 (80.3 mN/m), followed by NADES 10 (76.2 mN/m), both values being higher than that of the water (72.8 mN/m), corresponding to increased molecular adhesion and tighter surface structure.

In general, investigated NADESs show surface tension similar to that of DW and glycerols, and MI is considerably higher than that of MeOH (22.7 mN/m) and EtOH (22.4 mN/m). Hence, the investigated NADESs may possess both polar and nonpolar behavior depending on their hydrogen bond donor (HBD) and hydrogen bond acceptor (HBA) pairs.

#### 2.3.4. Polar Part of Surface Tension of NADESs

**Figure 1:**
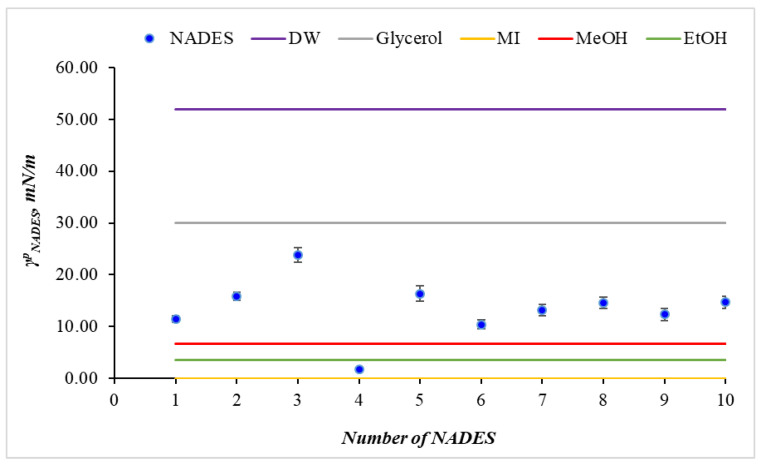
The position of NADES to classical solvents on the basis of their polar part of the surface tension. DW—distilled water; MI—methylene iodine.

The polar part, $\gamma_{NADES}^{p}$, is a key indicator of the strength of hydrogen bonds and dipole–dipole interactions in the NADES. Most values are in the range 10.4 to 23.8 mN/m (Figure 1, Table 3). NADES 4 is an exception, having much lower values of 1.8 mN/m. These results indicate the NADESs' pronounced ability to form hydrogen bonds, similar to glycerol (30.0 mN/m) but lower than that of DW (52.0 mN/m), whereas in NADES 4, the dominating Lifshitz–van der Waals interactions demonstrate the weak ability of NADES 4 to form specific interactions. Comparison with standard liquids demonstrates that the investigated NADES fill the area between nonpolar organic liquids and a highly polar water system, which determines their functional versatility.

#### 2.3.5. Polarity of NADESs

**Figure 2:**
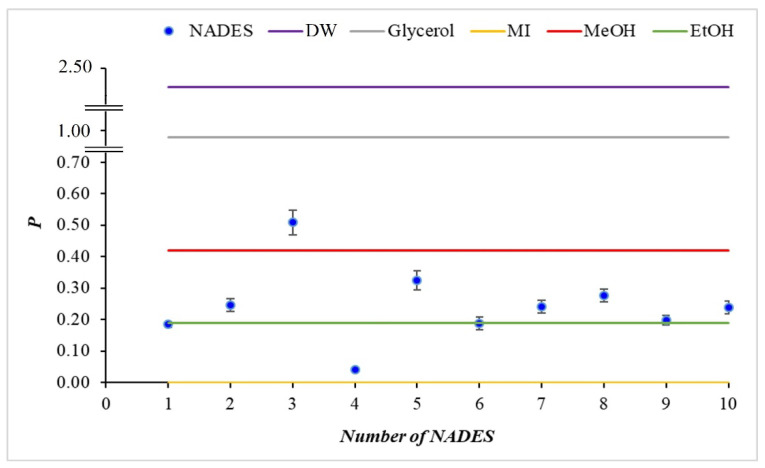
Polarity (P = $\gamma_{NADES}^{p}/\gamma_{NADES}^{d}$) for different NADESs, in comparison with some standard liquids. DW—distilled water; MI—methylene iodine.

Polarity, P, given as the ratio between the polar and dispersive parts of the surface tension $\gamma_{NADES}^{p}/\gamma_{NADES}^{d}$, is a measure of the relative contribution of the orientational and the dispersive forces in NADESs. For the highly polar DW, it is 2.34, and for glycerol, it decreases to 0.88, whereas for weakly polar liquids, such as EtOH and MI, it reaches values of 0.19 and 0.00, respectively (Figure 2 and Table 3).

According to the obtained *p*-values of NADES, ranging from 0.04 to 0.51, one can distinguish three different behaviors:

- High-polarity NADESs. This is NADES 3 (*p* = 0.51), having a value higher than that of MeOH but lower than that of glycerol. NADES 3 has a strongly polar character and can be classified as an adhesive system, showing strong interaction with surfaces and good wettability.
- Medium-polarity NADESs. For these NADESs, the polarity values are between those of EtOH and MeOH and correspond to NADES 2 and NADESs 5–10. They all have a pronounced polar character comparable to the standard solvents EtOH and MeOH.
- Low-polarity NADESs. Their values are lower than those of EtOH. NADES 4 (0.04) and NADES 1 (0.18) belong to this category. Those are cohesive systems, characterized by high internal stability and limited spreading on hard surfaces.

Most of the investigated NADESs (2, 5–10) belong to the medium polarity group, confirming that they have a balanced polar-dispersion profile that makes them compatible with both hydrophilic and hydrophobic substrates.

### 2.4. Solvatochromism

#### 2.4.1. Electron Transition Between Solvent and Solute

The polarity of the studied natural deep eutectic solvents (NADESs) was determined using the fluorescent indicator Nile Red, which allows for a reliable assessment of the microenvironment even in strongly acidic solvents, where the classic betaine 30 indicator is not applicable. The calculation of E_T_(30) was rendered infeasible due to the limited applications of the betaine 30 dye, its propensity to discolor in acidic environments, and the hypochromic shift in its absorption bands below 400 nm [89,90]. Consequently, the Nile Red dye was utilized, as it is unaffected by the pH of the environment. A number of earlier studies have established a correlation between E_T_(30) and E_NR_ [90,91]. Deye presents a graphical relationship between the two parameters, and later Cataldo establishes and reports a correlation between E_T_(30) and E_NR_ (Equation (17), Section 3.6) [90]. This facilitates the calculation of values on the E_T_(30) scale and the subsequent determination of normalized *E_T_^N^* values. This, in turn, enables direct comparison between different solvents, such as water and glycerol.

The E_NR_ values for NADES vary within a narrow range (48.4–49.4 kcal/mol), which indicates that all the mixtures studied provide a relatively homogeneous polar microenvironment for Nile Red (Table 4).

The range of the converted E_T_(30) values is from 59.01 to 62.04 kcal/mol, with the highest values being demonstrated by NADES 4 (U^1^G^6^MI^1^), NADES 5 (U^1^G^6^TA^1^), and NADES 10 (U^1^G^6^S^0.5^) (Table 4). This phenomenon can be attributed to the high acidity of the included carboxyl components, the substantial number of hydroxyl groups present in sucrose, and their capacity to engage in a substantial network of hydrogen bonds, thereby stabilizing more polar environments. Conversely, NADES 7–9 exhibit marginally diminished polarity (E_T_(30) ≈ 59 kcal/mol), likely attributable to the furanose ring of carbohydrates, which curtails the efficacy of hydrogen bonding (Table 4).

**Figure 3:**
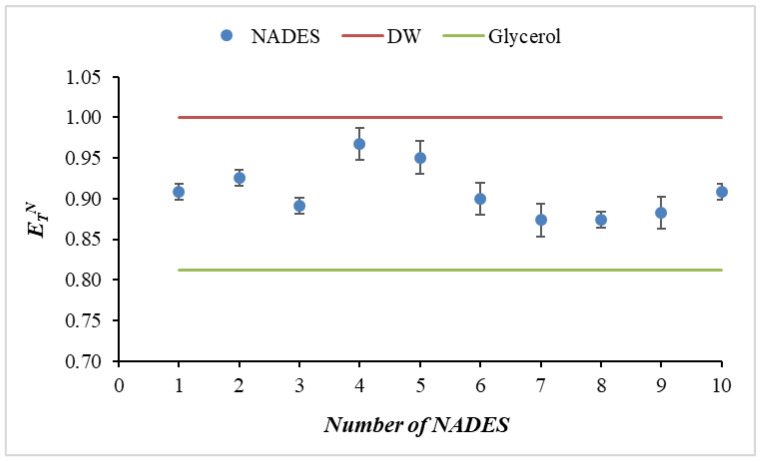
Normalized polarity (*E_T_^N^*) of the studied NADESs (1–10) compared with water and glycerol. DW—distilled water.

The *E_T_^N^* values obtained evidently delineate the NADESs between water (*E_T_^N^* ≈ 1.00) and glycerol (*E_T_^N^* ≈ 0.82) (Figure 3). This finding indicates that, despite substantial structural disparities, all NADESs under consideration can be categorized as highly polar solvents. The highest values (0.95–0.97) are reported for NADESs 4 and 5, which are close to the polarity of water (Table 4 and Figure 3).

Despite the presence of three carboxyl groups and one hydroxyl group, NADES containing citric acid (NADES 2) exhibits a lower polarity in comparison to systems containing maleic acid (NADES 4) and tartaric acid (NADES 5). This behavior may be attributed to the pronounced steric hindrance and strong intramolecular hydrogen bonds present in the citric acid molecule, which limit the accessibility of its functional groups for intermolecular interactions with urea. Consequently, the establishment of a substantial and well-organized network of hydrogen bonds in the liquid phase is impeded, resulting in a decline in polarity. In contrast, maleic and tartaric acids exhibit more linear and spatially accessible arrangements of carboxyl and hydroxyl groups, which facilitate more efficient intermolecular hydrogen bonds and greater structural order in the NADES matrix. Consequently, NADESs 4 and 5 form more open, dynamic, and interconnected networks of hydrogen bonds, which give higher E_T_(30) and *E_T_^N^* values. A similar trend is observed in the case of malonic acid. The two carboxyl groups are separated by a methylene group. The accumulation of carboxyl and hydroxyl groups leads to the creation of high local density, which hinders the access of urea to the functional groups. This indicates that while there are more groups, not all of them are effectively accessible for intermolecular interactions that create a polar environment. Conversely, NADESs 7 and 8 exhibit the lowest yet still significant polarity (*E_T_^N^* ≈ 0.87–0.88) (Figure 3). The presence of multiple hydroxyl and carboxyl groups in the composition of a NADES has been demonstrated to promote strong interfacial interactions and solvation of polar analytes. Furthermore, differences in polarity have been shown to be directly related to the number of available H-donor/acceptor centers and the steric characteristics of the organic components.

In summary, the analysis of *E_T_^N^* values demonstrates that through the judicious selection of HBD/HBA components, it is possible to meticulously adjust the polarity and solvation properties of deep eutectic solvents. This finding serves to substantiate the hypothesis that DES is a tunable, environmentally friendly, and highly efficient medium for the selective extraction of bioactive substances from plant materials.

#### 2.4.2. Kamlet−Taft Multiparameter Scale

The Kamlet–Taft parameters (*α*, *β*, and π*) are widely used to characterize specific and non-specific interactions, and they are a fundamental tool in the analysis of the structure, interactions, and functional properties of deep eutectic solvents (NADESs). The Kamlet–Taft multiparametric scale is a method that utilizes a variety of solvatochromic probes, including Nile red, Reichardt 30, 4-nitrophenol (NP), and 4-nitroanisol (ANS), to ascertain the values of disparate parameters for a given solvent [92,93]. The Kamlet–Taft parameters encompass dipolarity/polarizability (π*), hydrogen bond acidity (*α*), and hydrogen bond basicity (*β*), thus providing valuable insights into the properties of the solvent [37,39,89,94,95,96,97,98]. The Kamlet–Taft *α*, *β*, and π* parameters were determined for ten natural deep eutectic solvents (NADES), and their variations were analyzed to clarify the differences in the ability to donate/accept hydrogen bonds and in their dipolarity/polarizability. The values obtained manifest clearly expressed structure-property relationships, which are manifested as distinct trends in the series of solvents under consideration.

##### Acidity Parameter (α)

As illustrated in Table 4, the values for the multiparametric parameters of Kamlet–Taft are presented. The values of α range from 0.34 to 1.67, indicating a substantial variation in the capacity to donate a hydrogen bond between individual NADESs. In general, acidic NADESs have been found to exhibit high α values. Among them, NADES 4 (*α* = 1.67) is the most prominent, followed by NADES 5 (*α* = 1.49). NADESs containing citric and malonic acids (NADES 2 and 3) demonstrate a slight decrease in the same parameter (see Table 3). This finding demonstrates that the high local density of –COOH and –OH groups in the same acids impedes the transfer of H protons in the formation of hydrogen bonds with HBA. This finding is consistent with the results and the dependence obtained with the normalized *E_T_^N^* values (Figure 3). A comparison of all NADESs obtained revealed that NADES 4 (*α* = 1.67) exhibited the highest acidity, followed by NADES 10 (*α* = 1.54) and NADES 6 (*α* = 1.52) (Table 4). These systems are notable for their role as the strongest proton donors within the set. It is noteworthy that NADESs containing the aldose glucose (NADES 6) and the disaccharide sucrose (NADES 10) exhibit high α values when compared to the other acidic NADESs (NADESs 2, 3, and 5) (Table 4). This distinction may be attributed to the presence of a significant number of –OH groups. Fuad et al. report that α is predominantly influenced by the types of HBD [50]. As posited by Abbott et al., an excess of –OH groups has been demonstrated to enhance the strength of HBD [37]. In their investigation, Silva et al. examined the effects of DESs (type III) in the presence of choline chloride and carbohydrates, specifically monosaccharides (with furanose and pyranose rings) and the disaccharide sucrose. Their findings revealed that [Ch]Cl–glucose and [Ch]Cl–sucrose exhibited moderately high α values (1.15–1.27). These observations were attributed to the substantial number of hydroxyl groups present in sugars, which function as effective donors of hydrogen bonds. Furthermore, the same authors observed that [Ch]Cl-fructose marginally reduced the *α* parameter, while aldopentose exhibited the opposite effect, i.e., increased the α parameter [99]. In this particular case study, our focus is on a type-V NADES. In this instance, HBA is urea, which possesses a pronounced acceptor center. This feature may offer a rationale for the observation that NADES with glucose and sucrose exhibit elevated α values.

Conversely, NADES 8 (*α* = 0.34) and NADES 9 (*α* = 0.61) demonstrate a marked decline in acidity, signifying a limited capacity for hydrogen donation (Table 4). The graphical representation of *α* underscores the fact that the majority of NADESs exhibit moderate to high acidity, with NADES 8 and 9 representing the two clear outliers.

##### Basicness Parameter (β)

The *β* parameter demonstrates the widest dynamic range, from 0.10 to 4.41, indicating a broad spectrum of hydrogen bond acceptability among NADESs (Table 4). The highest *β* values were observed in NADES 10 (*β* = 4.41) and NADES 6 (*β* = 4.35), while NADES 1 (*β* = 3.26) also exhibited a distinctly basic character. Notably, NADES 6 and NADES 10 exhibited extremely high *β* values (4.35–4.41) (Table 4), suggesting the prevalence of strongly acceptor functional groups within these systems. This is likely attributable to the enhanced HBA functionality of the carbonyl group in urea. The β parameter is contingent on the type of acceptor; higher *β* values indicate enhanced proton acceptability, thereby facilitating the formation of H-bonds [50]. These observations indicate that the elevated *β* value in NADESs 6 and 10 does not result from the carbohydrate phase itself, but from the presence of strong HBA components in the eutectic structure, whose action is enhanced by the dense hydrogen network. The majority of other systems are distinguished by low basicity (*β* < 1.1), with NADES 3 (*β* = 0.10) and NADES 4 (*β* = 1.08) denoting the lower and middle limits, respectively. This finding suggests the presence of both strong and weak hydrogen bond acceptors within the set.

##### Dipolarity/Polarizability Parameter (π*)

The values of π* range from 0.33 to 2.10, indicating substantial disparities in the polarity of NADES systems (Table 4). NADES 8 (π* = 2.10) is distinguished as the most dipolar and polarizable system, followed by NADES 9 (π* = 1.70) and NADES 1 (π* = 1.47). Conversely, the π* value for NADESs 4, 6, and 10 is notably lower (π* = 0.33), indicating the presence of more strongly associated, “more compact” microdomains with restricted mobility and diminished capacity to solvate the probe molecules employed in solvatochromic analysis. This characteristic is likely attributable to reduced water content, a more robust internal hydrogen bond structure, and the potential for the formation of stable H-networks of hydrogen bonds with urea, which restrict the free polarizability of the system.

The implementation of correlation analysis has yielded a highly significant correlation between *α* and π* (r ≈ −0.99), thereby indicating that systems demonstrating a pronounced capacity for hydrogen donation tend to manifest reduced dipolarity and polarizability. This trend is clearly evident in NADES 4, which exhibits a high α value accompanied by a substantially lower π*, and similarly in NADES 8, which displays a contrasting profile.

##### Normalization of Kamlet–Taft Parameters—Assessment of NADES Polarity Relative to Classical Organic Solvents

**Figure 4:**
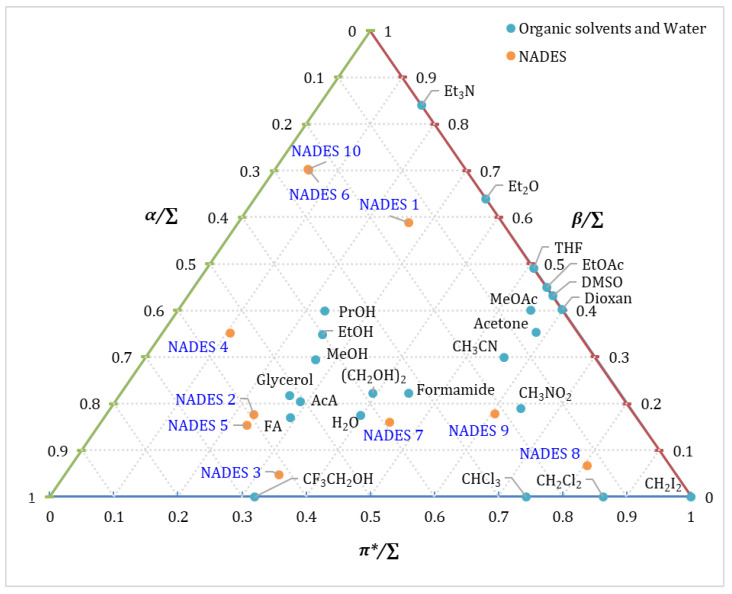
Comparative evaluation of Kamlet–Taft parameters of newly obtained NADES at 298 K with reference organic liquids. The reference normalized values *α/∑*, *β/∑*, and π*/∑ for organic solvents and water are taken from Snyder [100].

Natural deep eutectic solvents (NADESs) represent a new generation of “green” solvents that are the subject of intensive research due to their non-toxicity, biodegradability, and low cost. In order to optimize their application in chemical synthesis, catalysis, and extraction, it is necessary to precisely quantify their solvent properties. In the present study, the method of normalized Kamlet–Taft parameters (*α/∑*, *β/∑*, π*/∑) was applied to characterize 10 different NADESs and compare them with conventional organic solvents and water (Table S1 and Figure 4).

A detailed analysis of the positioning of NADES in the Kamlet–Taft triangle diagram, alongside their normalized values, reveals that they encompass an exceptionally broad and diverse spectrum of solvent properties in comparison to classical solvents, which are more confined in their distribution (Figure 4).

The prevailing parameter is the basis for the classification of NADES into three primary groups (A, B, and C). This classification is relative to the coordinates in the triangular diagram (i.e., the parameters of water *α/∑* = 0.43, *β/∑* = 0.18, π*/∑ = 0.43).

A. H-Acceptor Group (High Basicity, *β/∑*):

The group under discussion includes NADES 1, NADES 6, and NADES 10, which demonstrate the highest basicity values (*β/∑*, in the range 0.59–0.70). These solvents are positioned in the diagram in close proximity to strong Lewis bases (e.g., ethers and amines), surpassing most traditional polar aprotic solvents, such as DMSO, in terms of basicity (Figure 4). This renders them highly effective in the stabilization of protonic (acidic) solutes.

B. H-Donor Group (High Acidity, *α/∑*):

NADES 2, NADES 3, and NADES 5 are characterized by high acidity (*α/∑* in the di-range 0.59–0.62). Their position in close proximity to water, glycerol, and formic acid suggests that their potential as hydrogen bond donors is comparable to that of water (Figure 4). This profile has been demonstrated to be optimal for the strong solvation of basic solutes (H-acceptors). NADES 4 (*α/∑* = 0.54, *β/∑* = 0.35, π*/∑ = 0.11) is an H donor, but with high basicity. Its position is between the strongly acidic NADES 2, 3, 5, and the more moderate solvents such as ethanol (*β/∑* = 0.35) (Figure 4).

C. Dipole Group (High Polarizability, π*/∑):

NADES 8 and NADES 9 are dominated by dipolarity/polarizability (π*/∑ reaching 0.80 for NADES 8). These NADESs are grouped with halogenated solvents and other polar but weakly H-binding media. The solvation ability of these molecules is primarily attributable to their strong non-specific orientation and induction forces, rendering them suitable for the dissolution of highly polar molecules.

NADES 7 (*α/∑* = 0.39, *β/∑* = 0.16, π*/∑ = 0.45) is located in the central zone, showing a balanced solvation profile with moderate values of the three parameters (Figure 4). In addition, the normalized Kamlet–Taft parameters of the former are most similar to those of water (*α/∑* = 0.43, *β/∑* = 0.18, π*/∑ = 0.43). This position is analogous to that of classical proton solvents, but with a slight shift towards higher dipolarity.

The controlled arrangement of NADESs in the solvent space demonstrates the possibility of solvent “design.” For instance, while NADESs 3 and 5 are the strongest H donors, NADES 6 and 10 are the strongest H acceptors, thereby providing a solvent environment in which specific interactions are maximized in opposite directions. It is evident that NADESs are capable of covering areas that are otherwise inaccessible or difficult to achieve with a single conventional organic solvent.

## 3. Materials and Methods

### 3.1. Materials

Urea, glycerol, citric acid, malonic acid, maleic acid, tartaric acid, glucose, xylose, ribose, fructose, sucrose, Betaine 30, Nile red, 4-nitroanisole, and 4-nitrophenol were purchased from Sigma-Aldrich (St. Louis, MO, USA). All other chemicals were of analytical grade ≥ 99%.

### 3.2. Preparation of NADESs and Determination of Water Content

Urea, glycerol, citric, malonic, maleic, and tartaric acids, as well as glucose, fructose, sucrose, xylose, and ribose, were used after drying at 60 °C for 24 h and subsequent storage in a desiccator. Nine new ternary NADESs and one binary NADES were synthesized by weighing the specified molar ratios of HBA and HBD (1:6:1 and 1:2) into a synthesis vial placed under a nitrogen atmosphere to prevent moisture uptake from the air. The mixture was homogenized and heated at 80 °C in a glycerol bath at 800 rpm for 2–4 h until a transparent, homogeneous liquid was obtained. The reaction mixture was then allowed to cool to room temperature. The synthesized NADESs were left overnight to ensure that no precipitation formed [101]. The quantitative determination of the water content in the studied NADESs was performed using a moisture balance (MB45 Moisture Analyzer, NJ, USA). The analytical samples with a mass of 1 to 5 g were distributed in a uniform thin layer on an aluminum tray in order to optimize heat transfer and facilitate moisture desorption. A gradual heating regime was applied until the temperature range of 80–100 °C was reached. The selected temperature regime ensured complete evaporation of the water content while preventing thermal degradation of the NADES components. The duration of each analysis was fixed at 10 min, after which the process was automatically terminated when a constant mass was reached. In order to ensure statistical reliability, all measurements were performed in triplicate for each sample.

### 3.3. Determination of Physico-Chemical Properties

#### 3.3.1. Density

The densities of the NADESs were determined volumetrically using a pycnometer, previously calibrated with water at 25 °C, and an analytical balance with an accuracy of ±0.0001 g. The values were obtained from triplicate measurements and by calculating the density according to Equation (1), where *m* (g) is the mass and *V* (mL) is the volume of the NADES [102].

(7) $$\rho =\frac{m}{V}\;$$

#### 3.3.2. Refractive Index

The refractive index of the NADES was measured using an Abbe refractometer (RL-3, Warsaw, Poland) equipped with a thermostat for temperature control of the cell, with an accuracy of ±0.1 °C. Deionized water was used for calibration before each experiment. The standard uncertainty of the refractive index measurement on the $n_{D}$ scale was 0.0002. For each sample, at 25 °C, at least three independent measurements were performed to ensure reproducibility. The molar refraction (***R_m_***), free volume (***f_m_***), internal pressure (***P_int_***), and polarizability constant (***δ***) for NADESs were calculated by the following equations [103,104,105].

(8) $$M=\frac{X_{HBA\;}M_{HBA}+X_{HBD1\;}M_{HBD1}+X_{HBD2}M_{HBD2}}{X_{HBA\;}+X_{HBD1}+X_{HBD2}}$$

(9) $$R_{D}=\frac{n_{D}^{2}-1}{n_{D}^{2}+2}\cdot \frac{1}{\rho }$$

(10) $$R_{m}=\frac{n_{D}^{2}-1}{n_{D}^{2}+2}\;\cdot V_{m}=\frac{n_{D}^{2}-1}{n_{D}^{2}+2}\;\cdot \frac{M_{DES}}{\rho }$$

(11) $$R_{m}=\frac{4}{3}\pi N_{A\;}\delta$$

(12) $$\delta =\frac{3}{4}\cdot \frac{R_{m}}{\pi N_{A}}\;\;$$

(13) $$f_{m}=V_{m}-R_{m}$$

(14) $$P_{int}=\frac{2^{1/6\;}\;RT}{2^{1/6}\;V_{m}-d.N_{A}^{1/3}\;V_{m}^{2/3}}$$

(15) $$d=2{\left(\frac{{3\;R}_{m}}{4\;\pi N_{A}}\right)}^{1/3}$$

### 3.4. FTIR Spectral Analysis

FTIR spectra of the starting materials as well as of the eutectic solvents were recorded using a Bruker Alpha II infrared spectrometer (Bruker, Billerica, MA, USA). For each spectrum of the NADES and the starting components, 16 scans were performed with a selected resolution of 4.0 cm^−1^ over the range 400–4000 cm^−1^. Spectral acquisition was controlled using the Opus 8.7.41 software package. The spectra of the starting components were recorded in KBr pellets, where the sample was ground together with potassium bromide in an agate mortar to obtain a homogeneous powder, which was then pressed into a pellet. The FTIR spectra of the NADES were recorded by applying a thin film of the respective NADES onto an empty KBr pellet. Infrared spectroscopy (FTIR) was used to investigate the molecular interactions of NADESs. This technique helps identify the nature of interactions between the solute and the solvent, the presence of functional groups in the solvent mixture, and hydrogen bonds, which are a key feature of the structure of the NADES [106].

### 3.5. Surface Tension

As a first step, surface tensions of three smooth and clean solid surfaces, mainly stainless steel, microscopy glass, and a polymer plate, were obtained. The horizontal position of the solid plates was ensured by placing them on an optical table. Then, a small drop of the test liquid (2 μL volume) was carefully inserted onto the surface from a precise 10 micro syringe (Innovative Labor System GmbH, Stützerbach, Germany), supplied with a steel needle. The drop volume should not exceed 10 μL to prevent gravitational flattening of the drop. A USB microscope was used to capture the side view of each drop. The photos were used to determine the contact angles on the left and right sides of the drops, employing ImageJ v. 1.54f software.

DW and MI were used as test liquids. The experiments were performed at room temperature. For each solid plate, ten drops of each liquid were placed at different locations. The γ value for calculating surface tension is the average of all 20 contact angles, measured for a given liquid, at a particular solid. The deviations were less than 5%. The surface tension, with its polar and dispersive parts, of each solid was evaluated by analyzing Equation (3). The results are listed in Table 5. The values were then used as the NADES surface tension was being investigated.

The same procedure of placing small sessile drops on a solid surface was also used to determine the surface tension of NADES. In this case, the surface tension of the solid plate was known, so Equations (4)–(6) could then be employed to derive γ of the NADES.

### 3.6. Solvatochromism

To determine the empirical polarity of the new binary and ternary NADES, a spectrophotometer was used to record UV–Vis (M508, Leeds, UK) spectra and to identify the wavelength of maximum absorption. Solvatochromic probes—betaine 30, Nile Red (NR), 4-nitroanisole (ANS), and 4-nitrophenol (NP)—were used for the measurements. For the solvatochromic dyes, absorption was recorded in the range of 400–650 nm, while for ANS and NP, absorption was recorded in the range of 300–450 nm.

A stock solution with a concentration of 1.5 × 10^−3^ M in methanol is prepared from each probe. In total, 100 µL of the respective solution is dosed into glass tubes, after which the solvent is evaporated. A total of 3 mL of the NADES is added to the dry residue until a working concentration of 5 × 10^−5^ M is reached [89]. The mixtures are homogenized by heating in a water bath and using a vortex, during which the probes are completely dissolved within a few minutes.

These solvatochromic parameters can be calculated using the following equations, where *ν* is the wavenumber [90,92,93].

(16) $$E_{NR}=\frac{28,591}{\lambda_{max}},\;kcal/mol$$

(17) $$E_{T}\left(30\right)=\frac{E_{NR}-67.312}{-0.3039},kcal/mol\;$$

(18) $$E_{T}^{N}=\frac{E_{T}\left(30\right)-E_{T}(TMS)}{E_{T}\left(H_{2}O\right)-E_{T}(TMS)}=\frac{E_{T}\left(30\right)-30.7}{32.4}$$

(19) $$\alpha =\frac{19.9657-1.0241\pi^{*}-\upsilon_{NR}}{1.6078}$$

(20) $$\beta =\frac{1.0434\upsilon_{ANS}-0.57-\upsilon_{NP}}{2}$$

(21) $$\pi^{*}=\frac{\upsilon_{ANS}-34.12}{-2.4}$$

$$\upsilon =\frac{1}{\lambda_{max}10^{-4}}$$

### 3.7. Statistical Analysis

All solvatochromic and physicochemical analyses were performed in triplicate, while contact angle measurements for determining surface tension were conducted in ten replicates. Data are expressed as mean ± SD, and the level of statistical significance was set at *p* < 0.05.

## 4. Conclusions

This study demonstrates that the synthesized urea-based NADESs represent a structurally diverse and functionally tunable platform of green solvents. The physicochemical parameters clearly indicate that increasing the number of carboxyl and hydroxyl groups in the third component leads to more compact hydrogen-bonded networks, with higher density and reduced internal pressure, characteristic of less structured yet highly polar liquid media. FTIR analysis unambiguously confirms the absence of chemical reactions between the components and reveals the formation of stable intermolecular hydrogen bonds—the fundamental mechanism for DES structure formation.

Surface tension and contact angle measurements draw three clearly distinguishable groups of NADESs—highly polar, medium-polar, and low-polar systems. This diversity provides a unique advantage over conventional organic solvents, which occupy much more limited regions of the solvent space. Analysis of E_T_(30), *E_T_^N^*, and Kamlet–Taft parameters shows that polarity, acidity, and basicity can be precisely controlled by selecting an appropriate donor—carbohydrate, carboxylic acid, or glycerol. NADESs 3 and 5 stand out as the strongest H-bond donors, while NADESs 6 and 10 are dominated by H-bond accepting properties; NADESs 8 and 9 exhibit the highest dipolarity, making them suitable for specific applications requiring strong directional interactions.

Importantly, NADESs cover regions of solvent space inaccessible to any single class of organic solvents, demonstrating enormous potential for “design-on-demand.” This makes these systems highly promising for the selective extraction, dissolution, and stabilization of bioactive molecules, including phenolics, alkaloids, and other polar phytochemicals. Their combination of biodegradability, low toxicity, and the ability to finely tune solvation parameters positions these NADES as a promising basis for the future of green chemistry and sustainable technologies for natural product extraction.

## Figures and Tables

**Table 1:** Components of studied NADESs and water content.

№	Comp. 1	Comp. 2	Comp. 3	Molar Ratio	Abbr.	Water Content
						wt%
NADES 1	Urea	Glycerol	-	1:2	U^1^G^2^	1.35 ± 0.02
NADES 2	Urea	Glycerol	Citric acid	1:6:1	U^1^G^6^CA^1^	1.12 ± 0.03
NADES 3	Urea	Glycerol	Malonic acid	1:6:1	U^1^G^6^Mln^1^	1.92 ± 0.03
NADES 4	Urea	Glycerol	Maleic acid	1:6:1	U^1^G^6^Mlc^1^	1.84 ± 0.04
NADES 5	Urea	Glycerol	Tartaric acid	1:6:1	U^1^G^6^TA^1^	1.73 ± 0.02
NADES 6	Urea	Glycerol	Glucose	1:6:1	U^1^G^6^Glc^1^	1.82 ± 0.01
NADES 7	Urea	Glycerol	Xylose	1:6:1	U^1^G^6^X^1^	1.68 ± 0.05
NADES 8	Urea	Glycerol	Ribose	1:6:1	U^1^G^6^R^1^	1.72 ± 0.04
NADES 9	Urea	Glycerol	Fructose	1:6:1	U^1^G^6^F^1^	1.62 ± 0.01
NADES 10	Urea	Glycerol	Sucrose	1:6:1	U^1^G^6^S^0.5^	1.71 ± 0.04

**Table 2:** Physicochemical properties of studied NADESs—density (***ρ***), refractive index ($n_{D}$), molar refraction (***R_m_***), polarizability coefficient (***δ***), free volume (***f_m_***), and internal pressure (***P_int_***).

№	Abbr.	*ρ*	$n_{D}$	*R_m_*	*δ*, ×10^−24^	*f_m_*	*P_int_*
		g/cm^3^		cm^3^/mol			MPa
NADES 1	U^1^G^2^	1.243 ± 0.02	1.4795 ± 0.03	18.58 ± 0.21	7.37 ± 0.11	46.89 ± 0.21	138.44 ± 1.00
NADES 2	U^1^G^6^CA^1^	1.355 ± 0.05	1.4815 ± 0.01	21.15 ± 0.15	8.39 ± 0.08	53.10 ± 0.15	122.48 ± 1.23
NADES 3	U^1^G^6^Mln^1^	1.306 ± 0.03	1.4732 ± 0.02	19.25 ± 0.17	7.64 ± 0.09	49.35 ± 0.17	130.84 ± 1.93
NADES 4	U^1^G^6^Mlc^1^	1.315 ± 0.02	1.4780 ± 0.02	19.61 ± 0.23	7.78 ± 0.06	49.67 ± 0.23	130.54 ± 1.23
NADES 5	U^1^G^6^TA^1^	1.361 ± 0.03	1.4810 ± 0.01	19.96 ± 0.31	7.92 ± 0.18	50.17 ± 0.31	129.56 ± 1.05
NADES 6	U^1^G^6^Glc^1^	1.319 ± 0.02	1.4840 ± 0.03	20.69 ± 0.41	8.21 ± 0.23	51.62 ± 0.41	126.25 ± 0.99
NADES 7	U^1^G^6^X^1^	1.327 ± 0.04	1.4835 ± 0.01	21.34 ± 0.36	8.46 ± 0.21	53.32 ± 0.36	122.18 ± 1.12
NADES 8	U^1^G^6^R^1^	1.332 ± 0.02	1.4840 ± 0.01	20.48 ± 0.28	8.12 ± 0.16	51.10 ± 0.28	127.55 ± 1.65
NADES 9	U^1^G^6^F^1^	1.338 ± 0.02	1.4870 ± 0.03	21.30 ± 0.32	8.45 ± 0.18	52.75 ± 0.32	123.88 ± 1.41
NADES 10	U^1^G^6^S^0.5^	1.339 ± 0.03	1.4879 ± 0.02	22.49 ± 0.27	8.92 ± 0.16	55.58 ± 0.27	117.66 ± 1.05
H_2_O	*-*	0.997 ± 0.01	1.3330 ± 0.01	3.71 ± 0.08	1.47 ± 0.04	14.34 ± 0.08	395.27 ± 1.06
70% MeOH	*-*	0.950 ± 0.01	1.3415 ± 0.02	6.34 ± 0.11	2.52 ± 0.06	23.80 ± 0.11	240.14 ± 1.82

**Table 3:** Contact angles, *θ_NADES_*, surface tensions, *γ*_NADES_, with their polar, $\gamma_{NADES}^{p}$, and dispersive, $\gamma_{NADES}^{d}$ parts, and polarity, $P=\gamma_{NADES}^{p}/\gamma_{NADES}^{d}$, of investigated NADES and for surface tensions of some standard liquids. DW—distilled water; MI—methylene iodine.

DES	Abbr.	Med. Steel	Glass	Polymer	$\gamma_{NADES}^{d}$	$\gamma_{NADES}^{p}$	$\gamma_{NADES}$	*P*
		*θ_NADES_*, (°)			mN/m			
NADES 1	U^1^G^2^	65 ± 6	53 ± 3	70 ± 4	62 ± 5	11.39 ± 0.62	73 ± 4	0.184 ± 0.012
NADES 2	U^1^G^6^CA^1^	69 ± 4	54 ± 1	75 ± 2	64.3 ± 3.6	15.90 ± 0.74	80.3 ± 2.1	0.25 ± 0.02
NADES 3	U^1^G^6^Mln^1^	56 ± 1	45 ± 4	76 ± 2	46.89 ± 2.0	23.84 ± 1.46	70.7 ± 2.2	0.51 ± 0.04
NADES 4	U^1^G^6^Mlc^1^	49 ± 3	44 ± 2	41 ± 1	45.09 ± 0.31	1.77 ± 0.15	46.86 ± 0.30	0.041 ± 0.004
NADES 5	U^1^G^6^TA^1^	53 ± 3	46 ± 2	70 ± 6	50.4 ± 2.1	16.33 ± 1.48	67 ± 4	0.32 ± 0.03
NADES 6	U^1^G^6^Glc^1^	56 ± 3	51 ± 4	65 ± 4	55.3 ± 3.2	10.42 ± 0.82	65.7 ± 3.1	0.19 ± 0.02
NADES 7	U^1^G^6^X^1^	55 ± 4	51 ± 3	68 ± 2	55 ± 4	13.18 ± 1.05	67.9 ± 2.4	0.24 ± 0.02
NADES 8	U^1^G^6^R^1^	54 ± 5	49 ± 3	69 ± 3	52.7 ± 2.8	14.61 ± 1.14	67 ± 4	0.28 ± 0.02
NADES 9	U^1^G^6^F^1^	67 ± 4	53 ± 3	71 ± 2	62.6 ± 2.5	12.35 ± 1.17	74.9 ± 2.8	0.197 ± 0.015
NADES 10	U^1^G^6^S^0.5^	63 ± 4	56 ± 2	73 ± 3	62 ± 4	14.67 ± 1.20	76.2 ± 2.9	0.24 ± 0.02
**Referent Solvents** [79,82,87,88]								
					$\gamma_{l}^{d}$	$\gamma_{l}^{p}$	γ	$\gamma_{l}^{p}/\gamma_{l}^{d}$
					**mN/m**			
1	DW	-	-	-	21.8	51.0	72.8	2.34
2	Glycerol	-	-	-	34.0	30.0	64.0	0.88
3	MeOH	-	-	-	16.0	6.7	22.7	0.42
4	EtOH	-	-	-	18.8	3.6	22.4	0.19
5	MI	-	-	-	50.8	0.0	50.8	0.00

**Table 4:** Solvatochromic and Kamlet–Taft parameters (E_NR_, E_T_(30), *E_T_^N^*, *α*, *β*, π*) of the investigated NADESs (1–10).

№	Abbr.	E_NR_	E_T_(30)	*E_T_^N^*	*α*	*β*	π*
		kcal/mol					
NADES 1	U^1^G^2^	49.04 ± 0.03	60.12 ± 0.02	0.91 ± 0.01	0.81 ± 0.02	3.26 ± 0.01	1.47 ± 0.01
NADES 2	U^1^G^6^CA^1^	48.87 ± 0.03	60.67 ± 0.03	0.93 ± 0.01	1.43 ± 0.01	0.43 ± 0.02	0.56 ± 0.02
NADES 3	U^1^G^6^Mln^1^	49.21 ± 0.04	59.57 ± 0.02	0.89 ± 0.01	1.27 ± 0.02	0.10 ± 0.01	0.69 ± 0.02
NADES 4	U^1^G^6^Mlc^1^	48.46 ± 0.04	62.04 ± 0.04	0.97 ± 0.02	1.67 ± 0.03	1.08 ± 0.02	0.33 ± 0.01
NADES 5	U^1^G^6^TA^1^	48.62 ± 0.03	61.49 ± 0.04	0.95 ± 0.02	1.49 ± 0.02	0.37 ± 0.01	0.56 ± 0.01
NADES 6	U^1^G^6^Glc^1^	49.13 ± 0.04	59.84 ± 0.06	0.90 ± 0.02	1.52 ± 0.01	4.35 ± 0.01	0.33 ± 0.01
NADES 7	U^1^G^6^X^1^	49.38 ± 0.03	59.01 ± 0.04	0.87 ± 0.02	0.97 ± 0.02	0.40 ± 0.02	1.11 ± 0.02
NADES 8	U^1^G^6^R^1^	49.38 ± 0.04	59.01 ± 0.05	0.87 ± 0.01	0.34 ± 0.01	0.17 ± 0.01	2.10 ± 0.03
NADES 9	U^1^G^6^F^1^	49.29 ± 0.03	59.29 ± 0.06	0.88 ± 0.02	0.61 ± 0.02	0.50 ± 0.02	1.70 ± 0.01
NADES 10	U^1^G^6^S^0.5^	49.04 ± 0.03	60.12 ± 0.04	0.91 ± 0.01	1.54 ± 0.01	4.41 ± 0.03	0.33 ± 0.01

**Table 5:** Surface tension, *γ* of the three solid surfaces, together with its polar *γ*^p^ and dispersive *γ*^d^ parts.

Solid Surface	*γ* ^p^	*γ* ^d^	*γ*
	mN/m	mN/m	mN/m
stainless steel AISI 304 2B	38.7	14.7	53.4
microscopy glass	23.4	31.2	54.6
polymer plate	0.3	36.2	36.5

## Data Availability

Data are contained within the article and Supplementary Materials.

## Supplementary Materials

The following supporting information can be downloaded at: https://www.mdpi.com/article/10.3390/molecules31020233/s1. Figure S1: Changes in the FTIR spectral profile upon formation of the binary NADES U^1^G^2^ compared to the pure components urea and glycerol. The upper subscripts denote the molar ratio. Figure S2: Changes in the FTIR spectral profile upon formation of the ternary NADES U^1^G^6^CA^1^ compared to the pure components urea, glycerol, and citric acid. The upper subscripts denote the molar ratio. Figure S3: Changes in the FTIR spectral profile upon formation of the ternary NADES U^1^G^6^Mln^1^ compared to the pure components urea, glycerol, and malonic acid. The upper subscripts denote the molar ratio. Figure S4: Changes in the FTIR spectral profile upon formation of the ternary NADES U^1^G^6^Ml^1^ compared to the pure components urea, glycerol, and maleic acid. The upper subscripts denote the molar ratio. Figure S5: Changes in the FTIR spectral profile upon formation of the ternary NADES U^1^G^6^TA^1^ compared to the pure components urea, glycerol, and tartaric acid. The upper subscripts denote the molar ratio. Figure S6: Changes in the FTIR spectral profile upon formation of the ternary NADES U^1^G^6^Glc^1^ compared to the pure components urea, glycerol, and glucose. The upper subscripts denote the molar ratio. Figure S7: Changes in the FTIR spectral profile upon formation of the ternary NADES U^1^G^6^X^1^ compared to the pure components urea, glycerol, and xylose. The upper subscripts denote the molar ratio. Figure S8: Changes in the FTIR spectral profile upon formation of the ternary NADES U^1^G^6^R^1^ compared to the pure components urea, glycerol, and ribose. The upper subscripts denote the molar ratio. Figure S9: Changes in the FTIR spectral profile upon formation of the ternary NADES U^1^G^6^F^1^ compared to the pure components urea, glycerol, and fructose. The upper subscripts denote the molar ratio. Figure S10: Changes in the FTIR spectral profile upon formation of the ternary NADES U^1^G^6^S^0^.^5^ compared to the pure components urea, glycerol, and sucrose. The upper subscripts denote the molar ratio. Figure S11: Schematic representation of the balance between the tensions of each phase in contact (vapor, liquid, and solid), resulting in a contact angle (*θ*). Table S1: Normalized Kamlet–Taft parameters for the investigated NADESs (1–10). They are calculated based on the average values of the Kamlet–Taft parameters (α, β, and π*).
