# Supplementary material for: Solvatochromic Polarity, Physicochemical Properties, and Spectral Analysis of New Triple NADES-Based on Urea–Glycerol
Source: Molecules. 2026 Jan 9;31(2):233. doi: 10.3390/molecules31020233 (PMC12843706; doi:10.3390/molecules31020233)
Supplement: Supplementary file 1 [file molecules-31-00233-s001.zip › molecules-4069159-supplementary.pdf]

Solvatochromic polarity, physicochemical properties, and spectral analysis of new triple NADES based on urea–glycerol

Sezan Ahmed [1], Dimitar Bojilov [1]*, Ginka Exner [1], Soleya Dagnon [1], Stanimir Manolov [1], Iliyan Ivanov [1]

Contents

Figure S1. Changes in the FTIR spectral profile upon formation of the binary NADES U^1G^2 compared to the pure components urea and glycerol. The upper subscripts denote the molar ratio. 2

Figure S2. Changes in the FTIR spectral profile upon formation of the ternary NADES $U^1G^6CA^1$ compared to the pure components urea, glycerol and citric acid. The upper subscripts denote the molar ratio. 3

Figure S3. Changes in the FTIR spectral profile upon formation of the ternary NADES $U^1G^6Mln^1$ compared to the pure components urea, glycerol and malonic acid. The upper subscripts denote the molar ratio. 4

Figure S4. Changes in the FTIR spectral profile upon formation of the ternary NADES $U^1G^6Ml^1$ compared to the pure components urea, glycerol and maleic acid. The upper subscripts denote the molar ratio. 5

Figure S5. Changes in the FTIR spectral profile upon formation of the ternary NADES $U^1G^6TA^1$ compared to the pure components urea, glycerol and tartaric acid. The upper subscripts denote the molar ratio. 6

Figure S6. Changes in the FTIR spectral profile upon formation of the ternary NADES $U^1G^6Glc^1$ compared to the pure components urea, glycerol and glucose. The upper subscripts denote the molar ratio. 7

Figure S7. Changes in the FTIR spectral profile upon formation of the ternary NADES $U^1G^6X^1$ compared to the pure components urea, glycerol and xylose. The upper subscripts denote the molar ratio. 8

Figure S8. Changes in the FTIR spectral profile upon formation of the ternary NADES $U^1G^6R^1$ compared to the pure components urea, glycerol and ribose. The upper subscripts denote the molar ratio. 9

Figure S9. Changes in the FTIR spectral profile upon formation of the ternary NADES $U^1G^6F^1$ compared to the pure components urea, glycerol and fructose. The upper subscripts denote the molar ratio. 10

Figure S10. Changes in the FTIR spectral profile upon formation of the ternary NADES $U^1G^6S^{0.5}$ compared to the pure components urea, glycerol and sucrose. The upper subscripts denote the molar ratio. 11

Figure S11. Schematic representation of the balance between the tensions of each phase in contact (vapor, liquid and solid), resulting in a contact angle, $\boldsymbol{\theta}$. 11

Table S1. Normalized Kamlet–Taft parameters for the investigated NADES (1-10). They are calculated based on the average values of the Kamlet-Taft parameters (α, β, and $\pi*$) 12

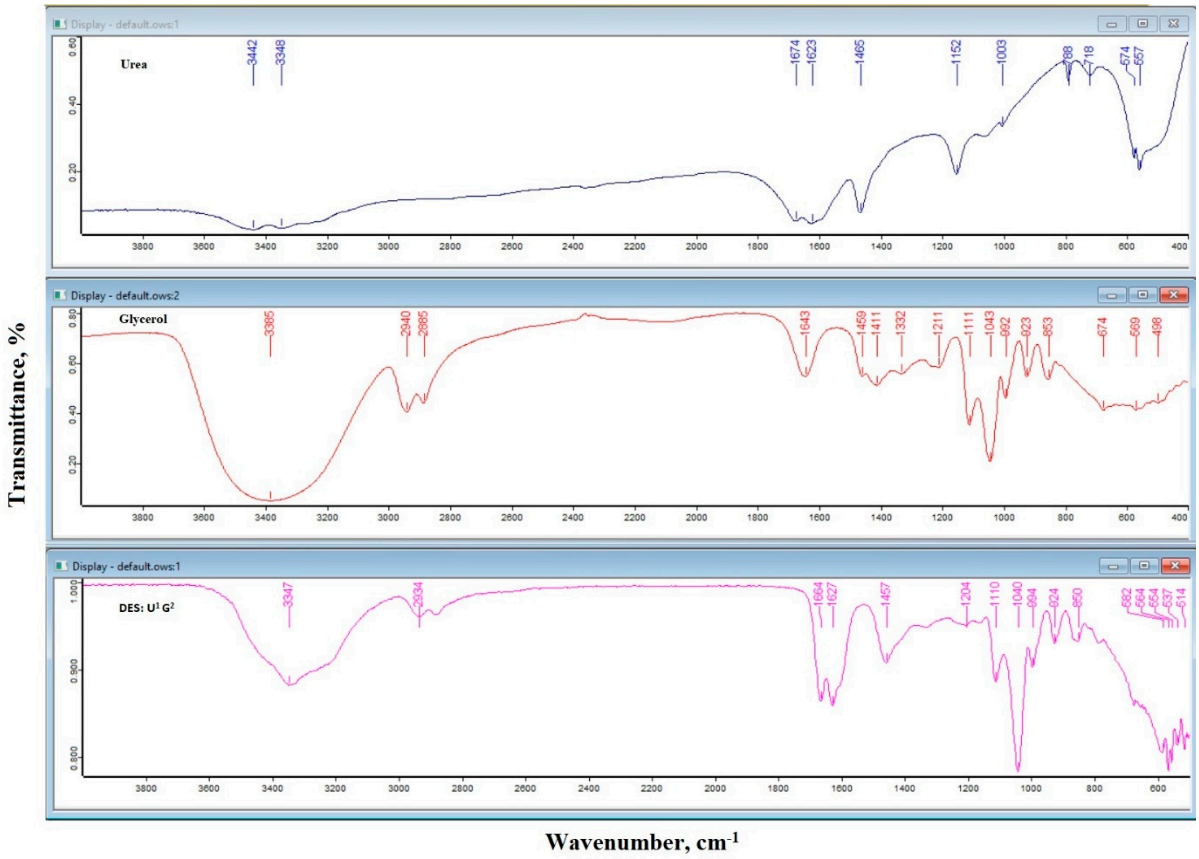

Figure S1. Changes in the FTIR spectral profile upon formation of the binary NADES U^1G^2 compared to the pure components urea and glycerol. The upper subscripts denote the molar ratio.

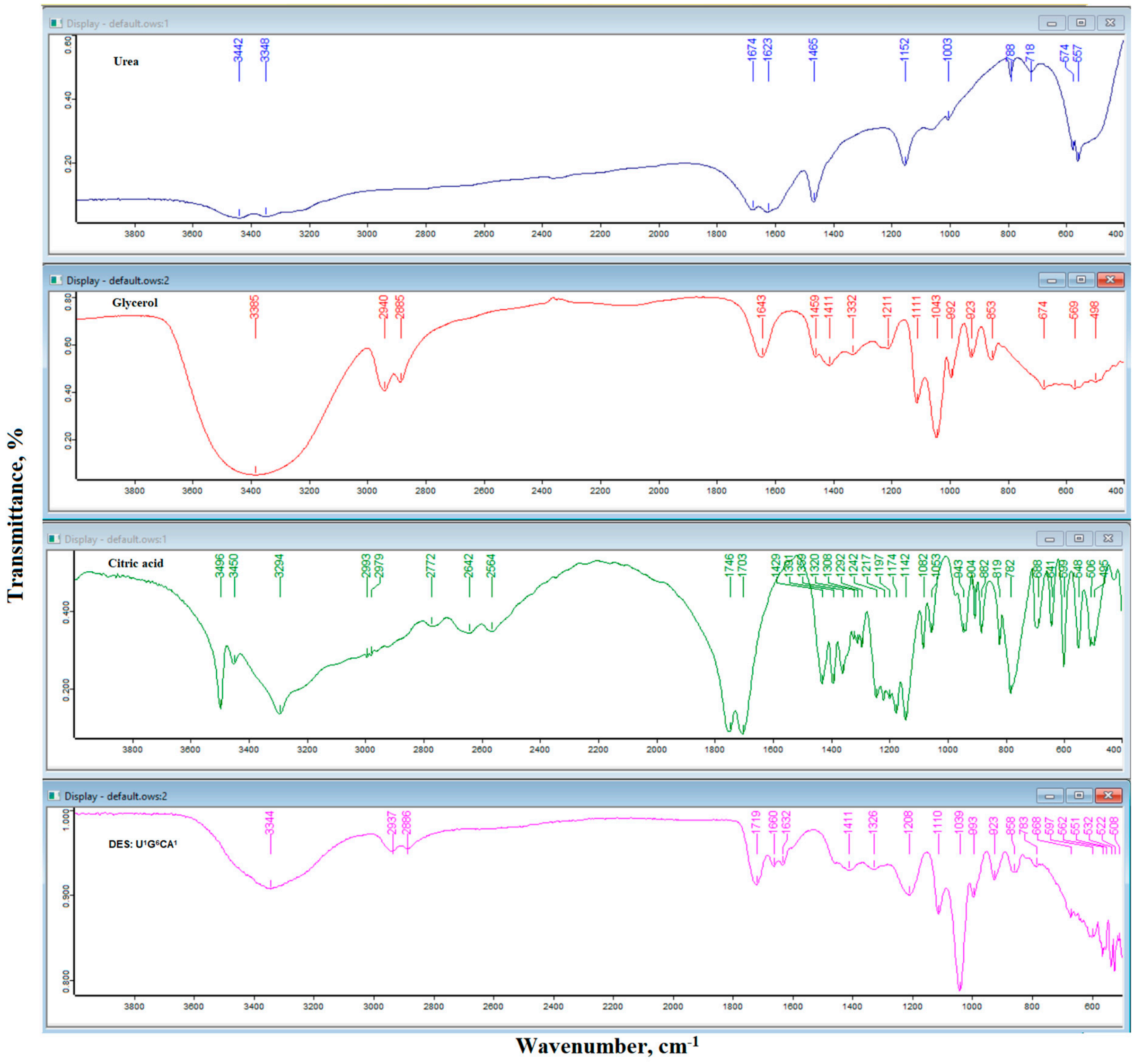

Figure S2. Changes in the FTIR spectral profile upon formation of the ternary NADES $U^1G^6CA^1$ compared to the pure components urea, glycerol and citric acid. The upper subscripts denote the molar ratio.

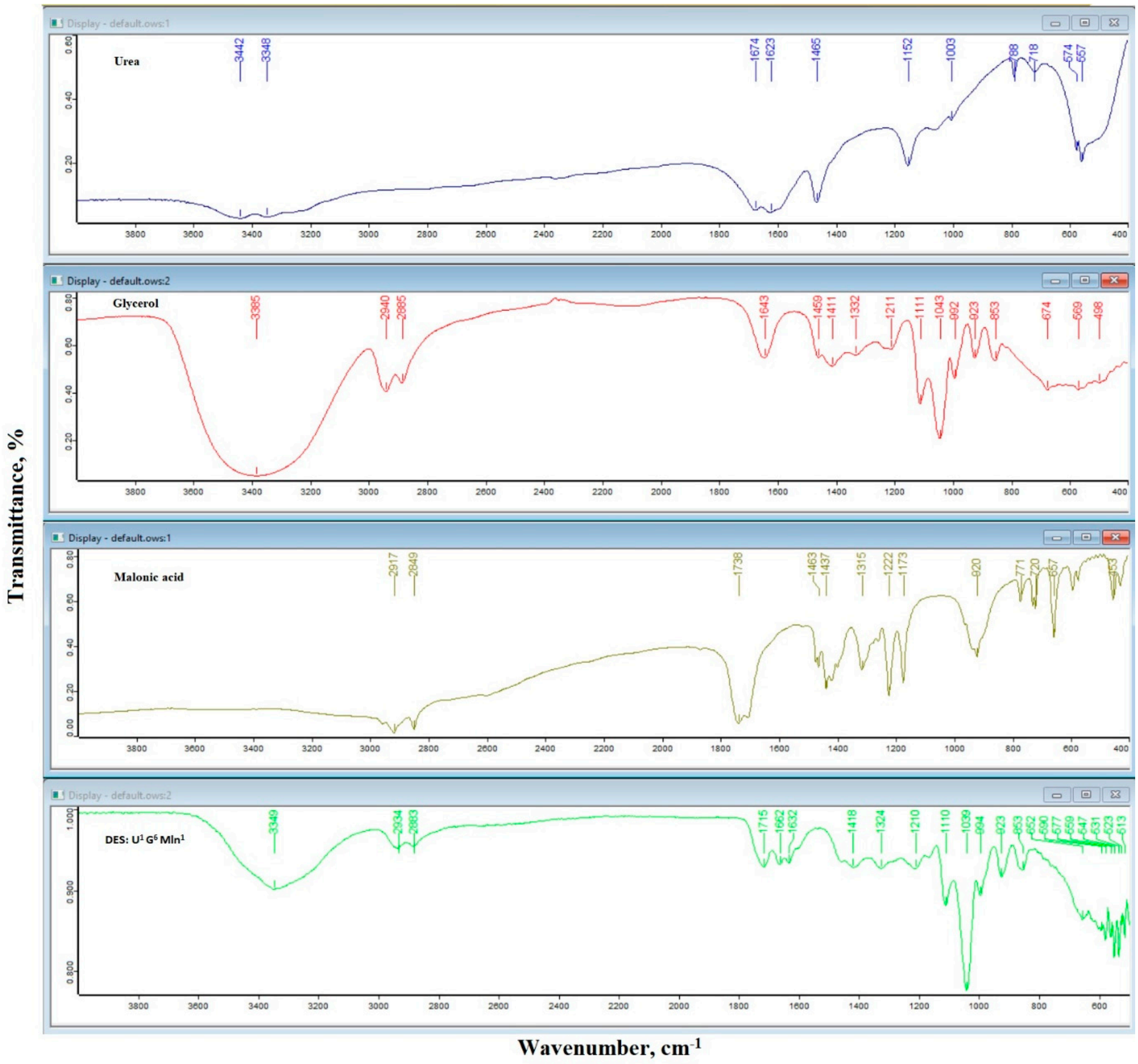

Figure S3. Changes in the FTIR spectral profile upon formation of the ternary NADES $U^1G^6Mln^1$ compared to the pure components urea, glycerol and malonic acid. The upper subscripts denote the molar ratio.

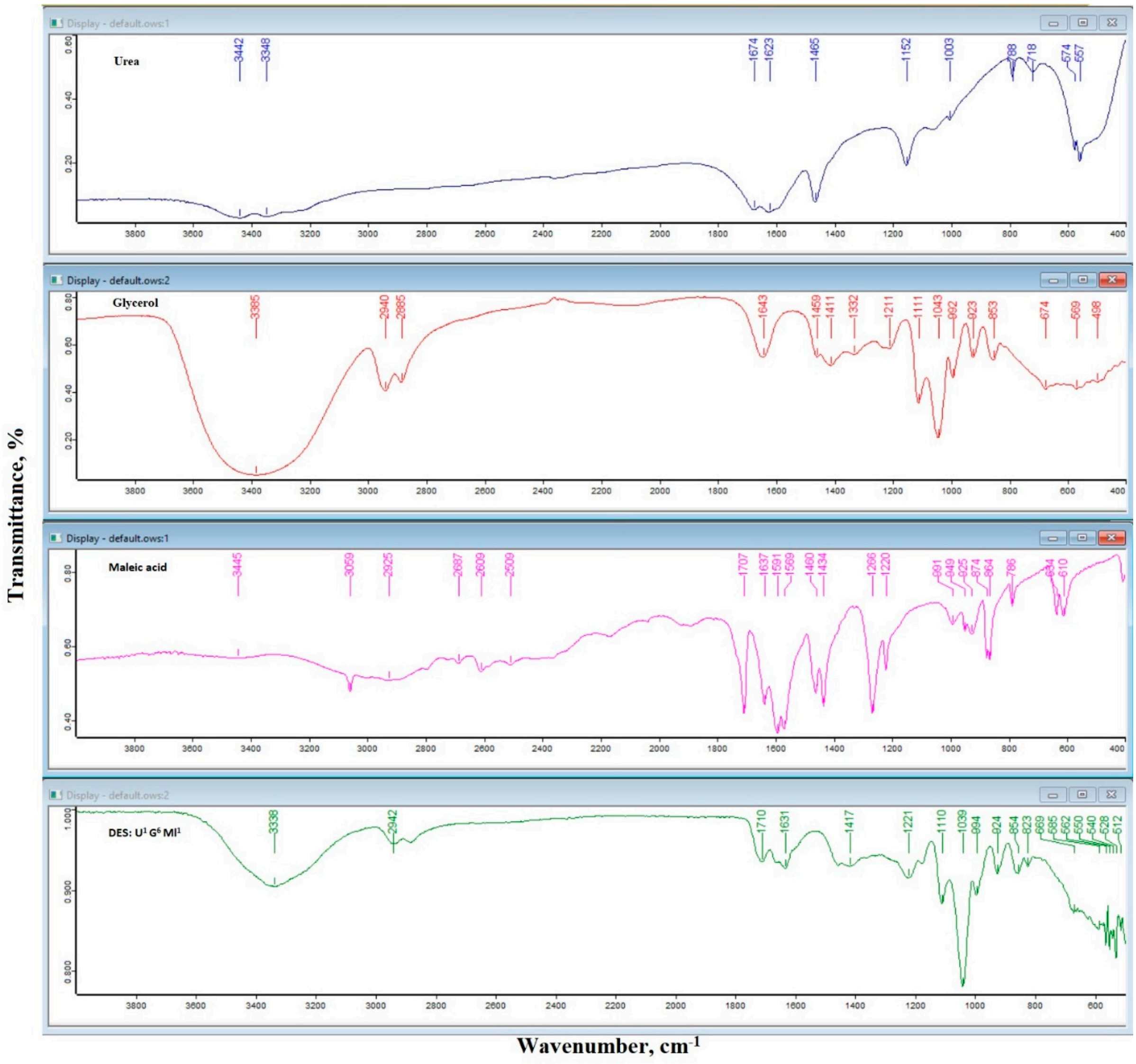

Figure S4. Changes in the FTIR spectral profile upon formation of the ternary NADES $U^1G^6Ml^1$ compared to the pure components urea, glycerol and maleic acid. The upper subscripts denote the molar ratio.

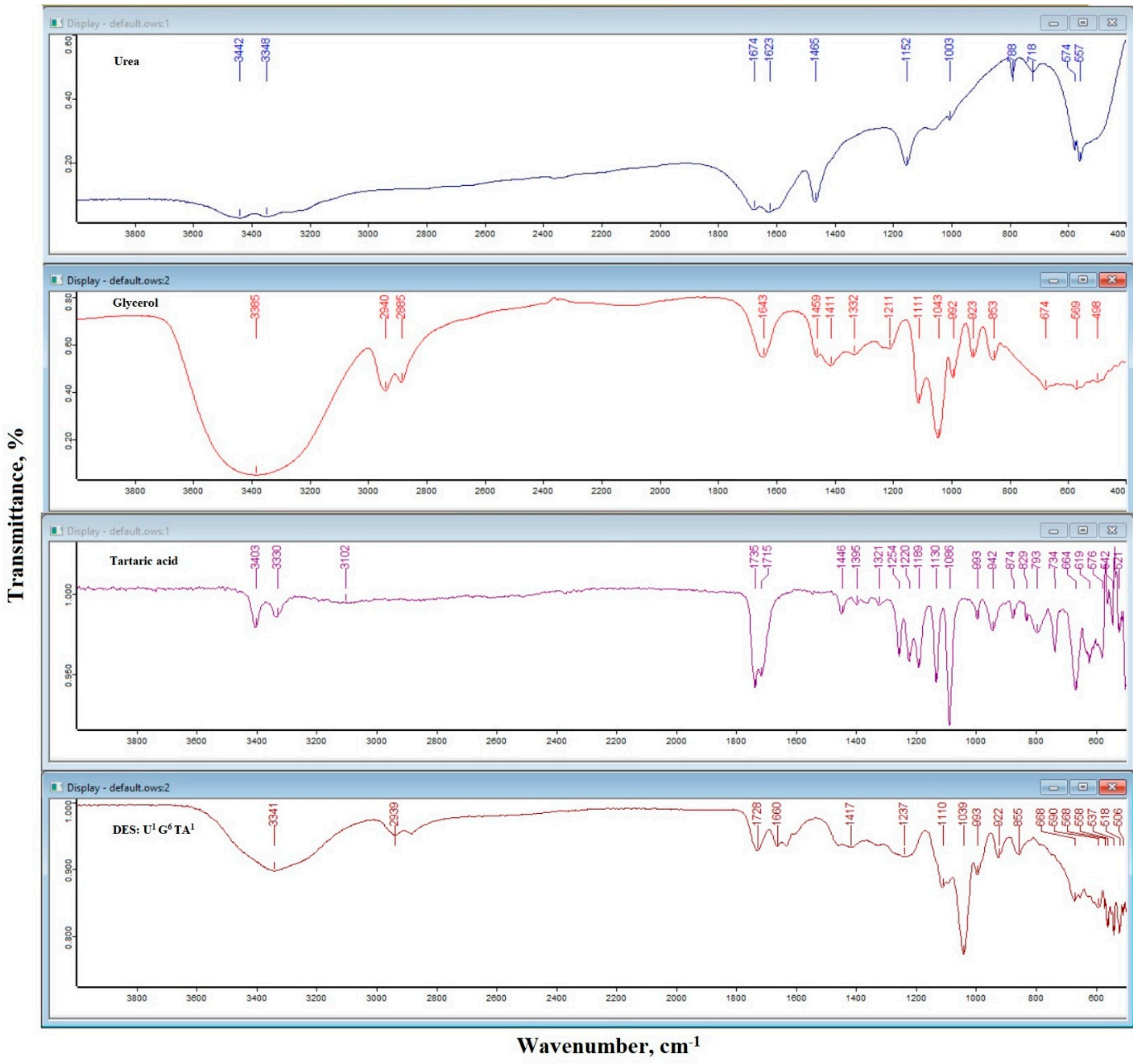

Figure S5. Changes in the FTIR spectral profile upon formation of the ternary NADES $U^1G^6TA^1$ compared to the pure components urea, glycerol and tartaric acid. The upper subscripts denote the molar ratio.

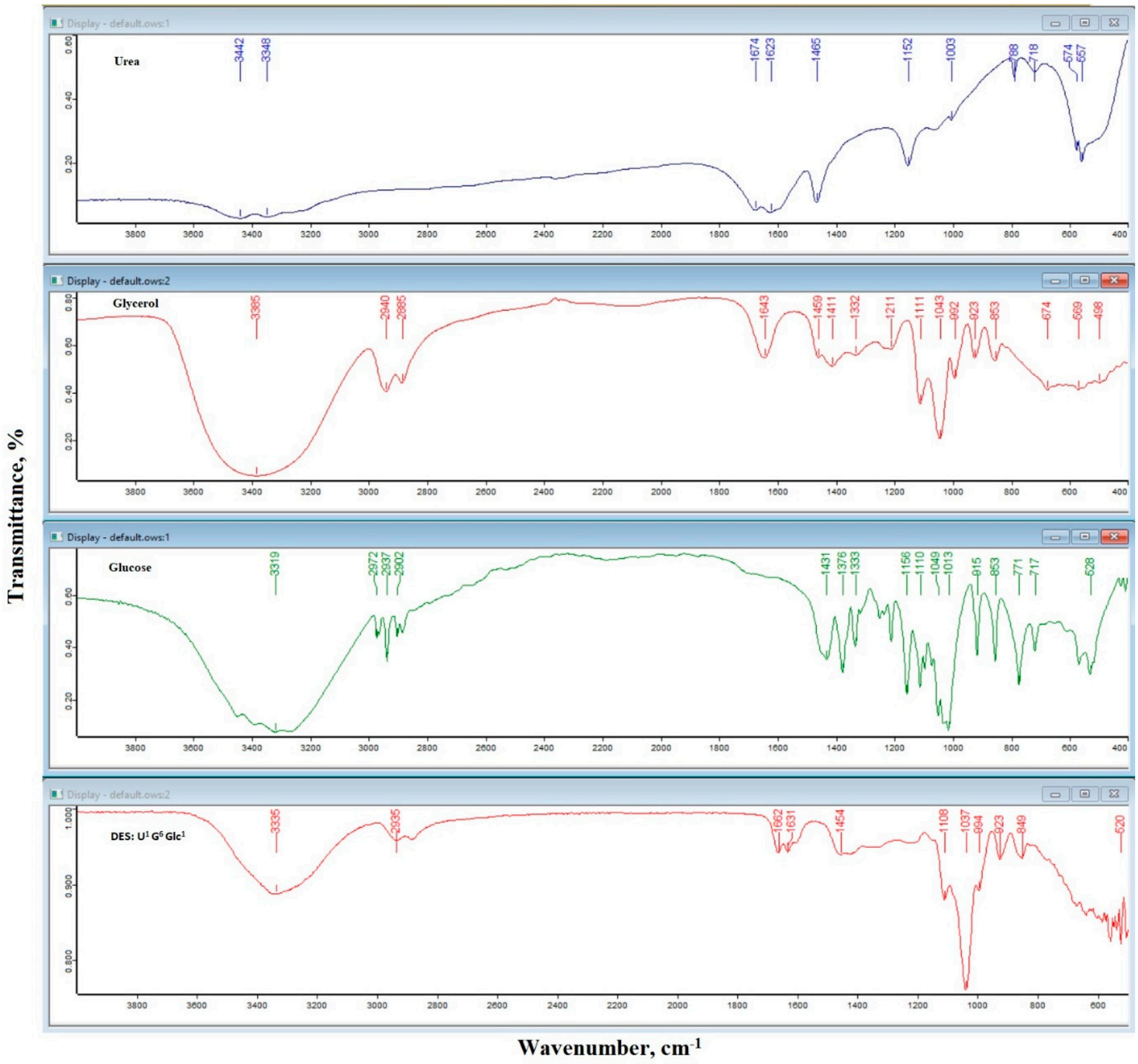

Figure S6. Changes in the FTIR spectral profile upon formation of the ternary NADES $U^1G^6Glc^1$ compared to the pure components urea, glycerol and glucose. The upper subscripts denote the molar ratio.

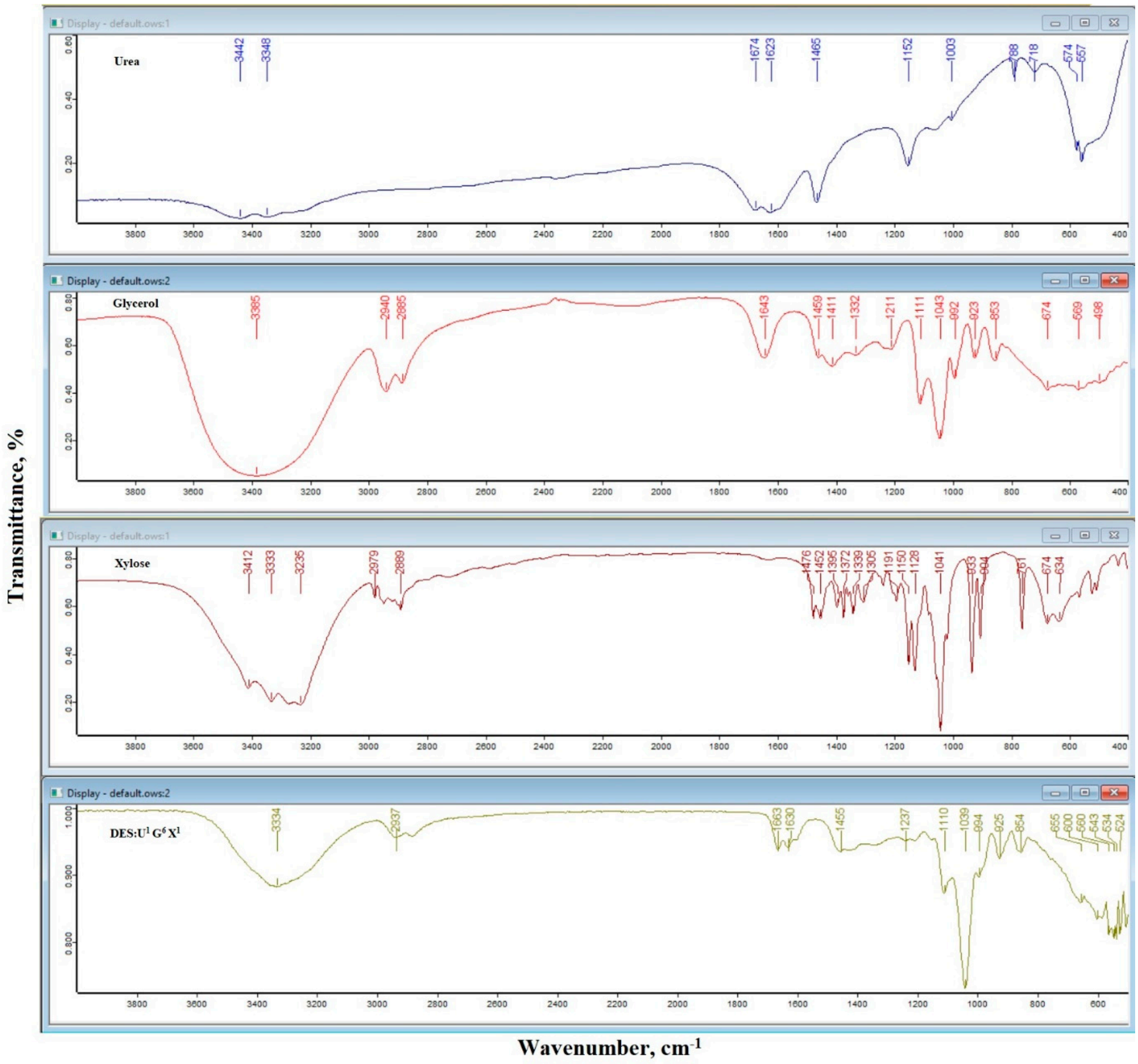

Figure S7. Changes in the FTIR spectral profile upon formation of the ternary NADES $U^1G^6X^1$ compared to the pure components urea, glycerol and xylose. The upper subscripts denote the molar ratio.

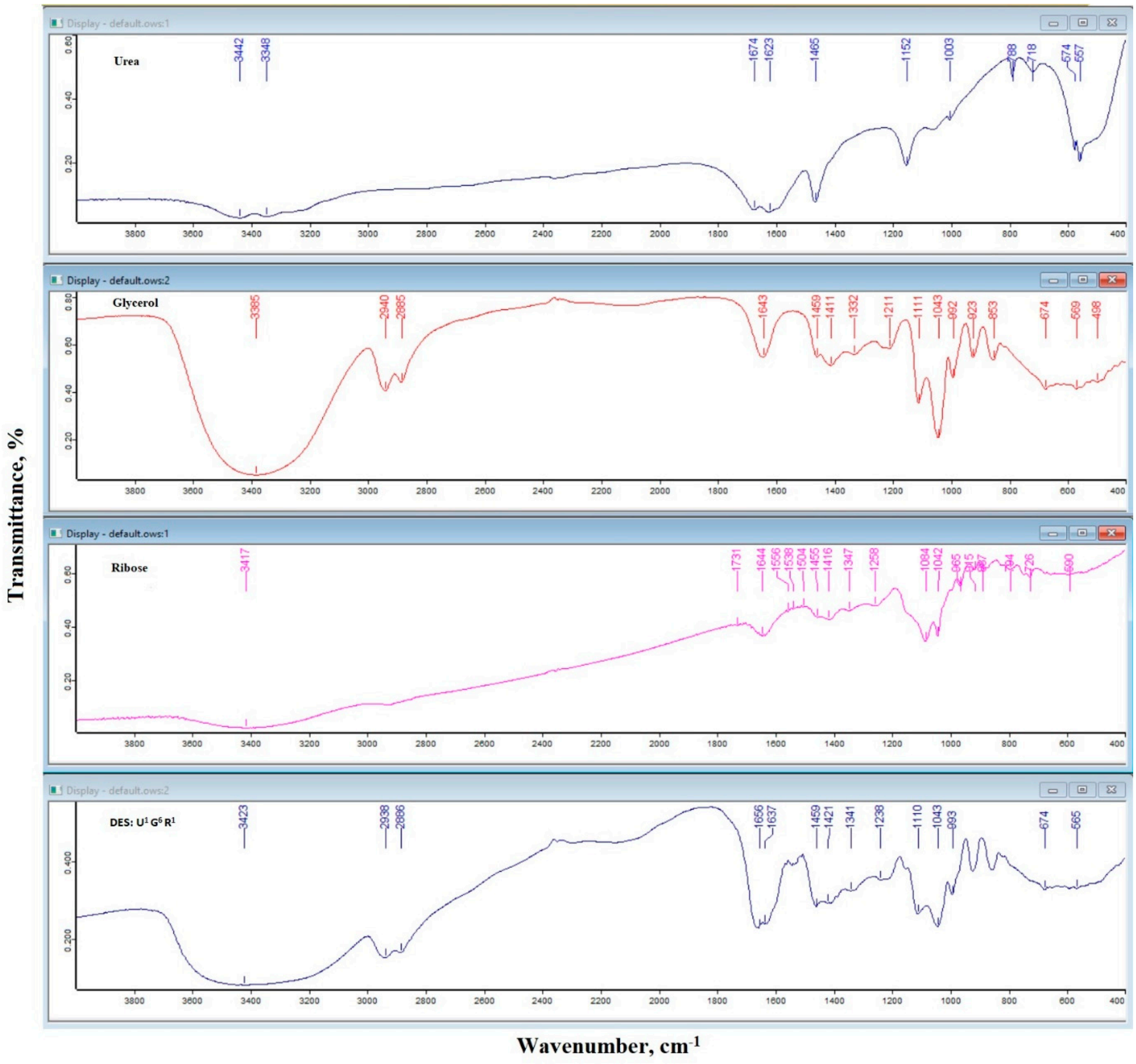

Figure S8. Changes in the FTIR spectral profile upon formation of the ternary NADES $U^1G^6R^1$ compared to the pure components urea, glycerol and ribose. The upper subscripts denote the molar ratio.

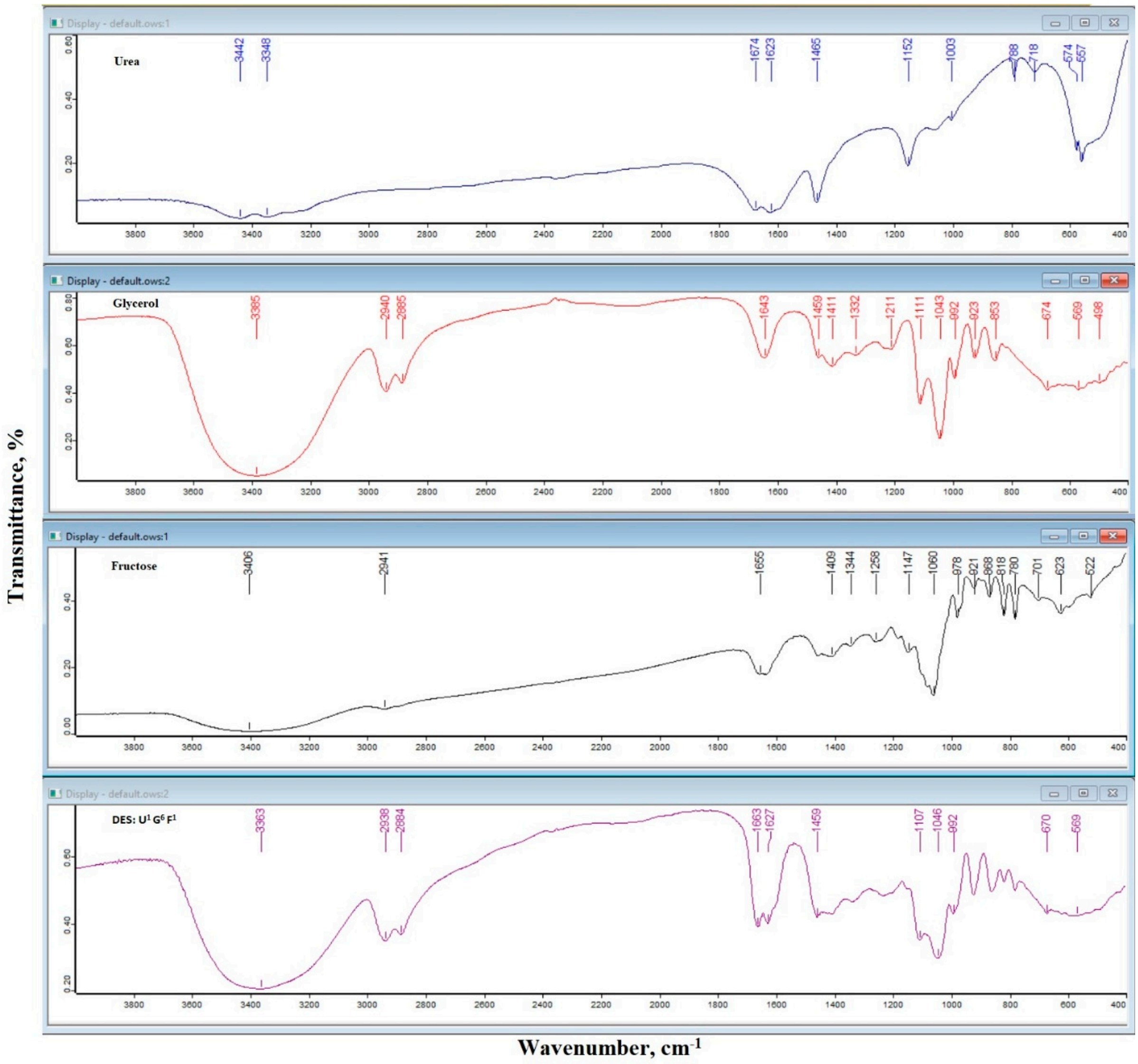

Figure S9. Changes in the FTIR spectral profile upon formation of the ternary NADES $U^1G^6F^1$ compared to the pure components urea, glycerol and fructose. The upper subscripts denote the molar ratio.

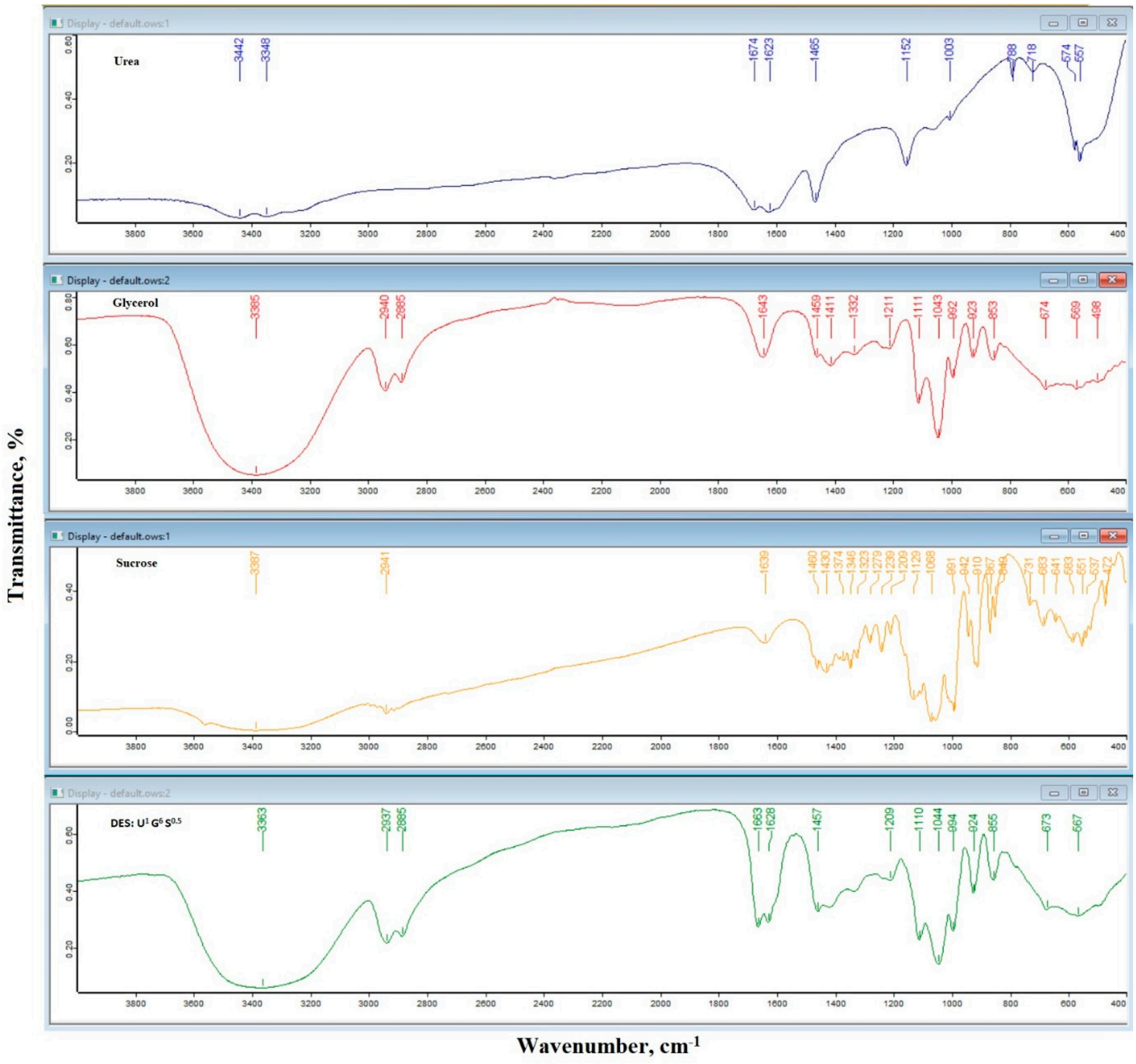

Figure S10. Changes in the FTIR spectral profile upon formation of the ternary NADES $U^1G^6S^{0.5}$ compared to the pure components urea, glycerol and sucrose. The upper subscripts denote the molar ratio.

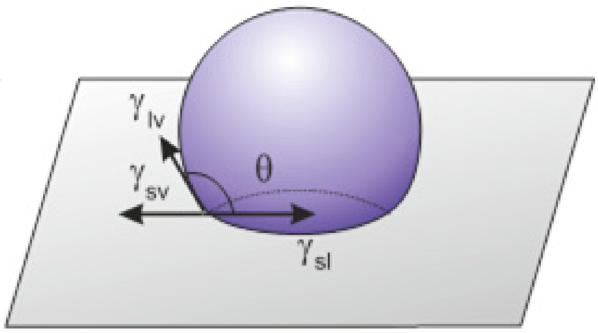

Figure S11. Schematic representation of the balance between the tensions of each phase in contact (vapor, liquid and solid), resulting in a contact angle, θ.

Table S1. Normalized Kamlet–Taft parameters for the investigated NADES (1-10). They are calculated based on the average values of the Kamlet-Taft parameters ($\alpha, \beta,$ and π^*)

**Normalized Kamlet-Taft parameters**		
$\boldsymbol{\alpha/\Sigma}$	$\boldsymbol{\beta/\Sigma}$	$\boldsymbol{\pi^*/\Sigma}$
0.15	0.59	0.27
0.59	0.18	0.23
0.62	0.05	0.33
0.54	0.35	0.11
0.62	0.15	0.23
0.25	0.70	0.05
0.39	0.16	0.45
0.13	0.07	0.80
0.22	0.18	0.61
0.25	0.70	0.05

$$\Sigma = \alpha + \beta + \pi^* \qquad \text{(S1)}$$

$$\alpha/\Sigma = \frac{\alpha}{\alpha + \beta + \pi^*} \qquad \text{(S2)}$$

$$\beta/\Sigma = \frac{\beta}{\alpha + \beta + \pi^*} \qquad \text{(S3)}$$

$$\pi^*/\Sigma = \frac{\pi^*}{\alpha + \beta + \pi^*} \qquad \text{(S4)}$$
